# Genome and evolution of the shade‐requiring medicinal herb *Panax ginseng*

**DOI:** 10.1111/pbi.12926

**Published:** 2018-05-25

**Authors:** Nam‐Hoon Kim, Murukarthick Jayakodi, Sang‐Choon Lee, Beom‐Soon Choi, Woojong Jang, Junki Lee, Hyun Hee Kim, Nomar E. Waminal, Meiyappan Lakshmanan, Binh van Nguyen, Yun Sun Lee, Hyun‐Seung Park, Hyun Jo Koo, Jee Young Park, Sampath Perumal, Ho Jun Joh, Hana Lee, Jinkyung Kim, In Seo Kim, Kyunghee Kim, Lokanand Koduru, Kyo Bin Kang, Sang Hyun Sung, Yeisoo Yu, Daniel S. Park, Doil Choi, Eunyoung Seo, Seungill Kim, Young‐Chang Kim, Dong Yun Hyun, Youn‐Il Park, Changsoo Kim, Tae‐Ho Lee, Hyun Uk Kim, Moon Soo Soh, Yi Lee, Jun Gyo In, Heui‐Soo Kim, Yong‐Min Kim, Deok‐Chun Yang, Rod A. Wing, Dong‐Yup Lee, Andrew H. Paterson, Tae‐Jin Yang

**Affiliations:** ^1^ Department of Plant Science, Plant Genomics and Breeding Institute Research Institute of Agriculture and Life Sciences College of Agriculture and Life Sciences Seoul National University Seoul Korea; ^2^ Phyzen Genomics Institute Seongnam Gyeonggi‐do Korea; ^3^ Department of Life Science Chromosome Research Institute Sahmyook University Seoul Korea; ^4^ Bioprocessing Technology Institute Agency for Science, Technology and Research (A*STAR) Singapore City Singapore; ^5^ School of Chemical Engineering Sungkyunkwan University Jangan‐gu, Suwon, Gyeonggi‐do Korea; ^6^ College of Pharmacy and Research Institute of Pharmaceutical Science Seoul National University Seoul Korea; ^7^ Department of Organismic and Evolutionary Biology Harvard University Herbaria Cambridge MA USA; ^8^ Planning and Coordination Division NIHS, RDA Wanju‐gun Jeollabuk‐do Korea; ^9^ Ginseng Research Division National Institute of Horticultural & Herbal Science, RDA Eumseong Chungcheongbuk‐do Korea; ^10^ Department of Biological Sciences Chungnam National University Daejeon Korea; ^11^ Department of Crop Science Chungnam National University Daejeon Korea; ^12^ Genomics Division National Institute of Agricultural Sciences Jeonju Jeollabuk‐do Korea; ^13^ Department of Bioindustry and Bioresource Engineering Plant Engineering Research Institute Sejong University Seoul Korea; ^14^ Division of Integrative Bioscience and Biotechnology Sejong University Seoul Korea; ^15^ Department of Industrial Plant Science & Technology Chungbuk National University Cheongju Chungcheongbuk‐do Korea; ^16^ Laboratory of Resource and Analysis R&D Headquarters Korea Ginseng Corporation Daejeon Korea; ^17^ Department of Biological Sciences College of Natural Sciences Pusan National University Busan Korea; ^18^ Korean Bioinformation Center Korea Research Institute of Bioscience and Biotechnology Daejeon Korea; ^19^ Graduate School of Biotechnology and Ginseng Bank Kyung Hee University Yongin Gyeonggi‐do Korea; ^20^ Arizona Genomics Institute School of Plant Sciences The University of Arizona Tucson AZ USA; ^21^ Plant Genome Mapping Laboratory College of Agricultural and Environmental Sciences and Franklin College of Arts and Sciences University of Georgia Athens GA USA

**Keywords:** *Panax ginseng*, ginsenosides, evolution, metabolic network, adaptation

## Abstract

*Panax ginseng* C. A. Meyer, reputed as the king of medicinal herbs, has slow growth, long generation time, low seed production and complicated genome structure that hamper its study. Here, we unveil the genomic architecture of tetraploid *P. ginseng* by *de novo* genome assembly, representing 2.98 Gbp with 59 352 annotated genes. Resequencing data indicated that diploid *Panax* species diverged in association with global warming in Southern Asia, and two North American species evolved via two intercontinental migrations. Two whole genome duplications (WGD) occurred in the family Araliaceae (including *Panax*) after divergence with the Apiaceae, the more recent one contributing to the ability of *P. ginseng* to overwinter, enabling it to spread broadly through the Northern Hemisphere. Functional and evolutionary analyses suggest that production of pharmacologically important dammarane‐type ginsenosides originated in *Panax* and are produced largely in shoot tissues and transported to roots; that newly evolved *P. ginseng* fatty acid desaturases increase freezing tolerance; and that unprecedented retention of chlorophyll a/b binding protein genes enables efficient photosynthesis under low light. A genome‐scale metabolic network provides a holistic view of *Panax* ginsenoside biosynthesis. This study provides valuable resources for improving medicinal values of ginseng either through genomics‐assisted breeding or metabolic engineering.

## Introduction

Roots of Asian/Korean ginseng have been used for thousands of years, today being an important Asian agricultural commodity with markets (together with *P. quinquefolius*, American ginseng) estimated at over 2 billion USD (Baeg and So, 2013). *Panax* species are shade‐requiring perennials (Court, 2000). Most diploid *Panax* such as *P. notoginseng*,* P. vietnamensis, P. bipinnatifidus, P. stipuleanatus* and *P. pseudoginseng* grow at high altitudes in warm freeze‐free areas from the Eastern Himalayas through Southern China to north and central highlands of Vietnam. Tetraploid *P. ginseng* and *P. quinquefolius* overwinter and are broadly distributed in Northeast Asia and North America, respectively.

Therapeutic effects of *P. ginseng* on neurodegenerative disorders (Cho, 2012; Radad *et al*., 2006), cardiovascular diseases (Zheng *et al*., 2012), diabetes (Xie *et al*., 2005) and cancer (Jung *et al*., 2012; Wong *et al*., 2015) are often attributed to unique saponins called ginsenosides, glycosylated triterpenes classified as either dammarane‐ (*Panax*‐specific) or oleanane‐type based on aglycone skeletal structure. Ginsenosides are accumulated in roots, leaves, stems, flower buds and berries, in quantities varying with tissue (Oh *et al*., 2014; Shi *et al*., 2007), age (Shi *et al*., 2007; Xiao *et al*., 2015), environment (Jiang *et al*., 2016; Kim *et al*., 2014) and cultivar (Lee *et al*., 2017b). Limited genomic resources and genetic populations due to slow growth (~4 years/generation), sensitivity to environmental stresses and low seed yield (40/generation) hamper developmental and genetic studies and breeding. Therefore, less numbers of ginseng cultivars were developed and those cultivars were not pure inbred line, containing some heterogeneity because seeds were multiplied by pedigree selection.

Here, we report a draft genome sequence of *P. ginseng* cultivar (cv.) Chunpoong (ChP), which is the first cultivar officially registered in Korea Seed and Variety Service and showed relatively uniform genotypes (Kim *et al*., 2012). Investigation of the *P. ginseng* genome and comparative analyses with carrot (*Daucus carota*; Iorizzo *et al*., 2016) and other plants allowed us to gain new insights into evolution and speciation, also clarifying the origin and regulation of ginsenoside accumulation. These discoveries provide a valuable foundation for improving therapeutic effects, understanding shade plant biology and empowering Araliaceae genomic studies.

## Results

### Genome assembly and annotation

Paired‐end (PE) reads covering 746 Gbp (206×) and mate‐pair (MP) reads covering 365 Gbp (101×) from ChP (Table S1) were assembled into 9,845 scaffolds covering 2.98 Gbp with N50 of 569 Kbp and longest scaffold of 3.6 Mbp (Table 1; Table S2). The predicted *P. ginseng* genome size ranged from 3.3 to 3.6 Gbp through flow cytometry and *k*‐mer frequency, slightly bigger than the reported 3.12 Gbp (Hong *et al*., 2004). Assembly accuracy and completeness was indicated by correct read mapping of four MP libraries revealing proper span size (Table S3); alignment to 13 finished bacterial artificial chromosome (BAC) sequences (Choi *et al*., 2014; Jang *et al*., 2017) showing 99% homology with perfect contiguity (Table S4); and Benchmarking Universal Single‐Copy Orthologs (BUSCO_v2) analysis finding 1339 (93%) of 1440 conserved orthologous angiosperm genes assembled completely (Table S5). Evidence‐based *de novo* annotation revealed 2181 Mbp (79.52%) of repetitive elements (REs) including long terminal repeat retrotransposons (LTR‐RTs) being most abundant with LTR/Gypsy accounting for 49% and one *PgDel* family alone occupying 30% of the genome (Table S6).

**Table 1:** *Panax ginseng* genome assembly and gene annotation parameters

Genome assembly	
Number of scaffolds	9845
Total length of scaffolds (bp)	2 984 993 682
N50 of scaffold (bp)	569 017
Longest scaffold (bp)	3 641 815
GC content (%)	32
Gene annotation	
Number of genes	59 352
Total coding sequence length (bp)	66 481 566
Mean gene length (bp)	4394
Mean number of exon per gene	5
Mean exon length (bp)	242
Average CDS length (bp)	1120
Maximum gene length (bp)	93 383
Average intergenic region length (bp)	37 601
Number of long noncoding RNAs (lncRNAs)	19 495
Number of conserved miRNAs	451

Using 104 Gbp of RNA‐Seq data (Table S7) and 184 171 PacBio transcripts, Integrated Pipeline for Genome Annotation (IPGA) (Figure S1) predicted 59 352 protein‐coding genes with average 1120 bp length, 86% supported by Illumina RNA‐Seq data (Jayakodi *et al*., 2018). *P. ginseng* genes showed average length and number of coding sequence (CDS) similar to other plants (Figure S2). The longest gene was 93 kb after manual curation based on PacBio transcripts (Table S8). Overall, 97% of *P. ginseng* transcripts have functional descriptions, with matches to known proteins in InterPro (84%), NCBI Nr (95%), Arabidopsis (89%) and tomato (92%) (Table S9). Approximately 82% of genes were associated with Gene Ontology (GO) functional classifications with >20% involved in biosynthetic pathways. Alternatively spliced (AS) transcripts were identified for 38% (22 384) of annotated genes, with intron retention the most common (Figure S3) AS type, consistent with other plants (Barbazuk *et al*., 2008). AS containing genes were enriched in sugar‐related metabolic process such as glycolytic process, response to fructose and starch biosynthetic process (Table S10). We predicted 19 495 long noncoding RNAs (lncRNAs) and 451 conserved micro RNAs (miRNAs; Table 1); 3588 transcription factors (TFs) (6.05% of annotated genes); 851 transcriptional regulators (1.43%); and 2209 protein kinases (3.72%), 1.6–2.0 times more than 18 plant genomes compared (Tables S11–S13).

### Genome structure and evolution

**Figure 1:**
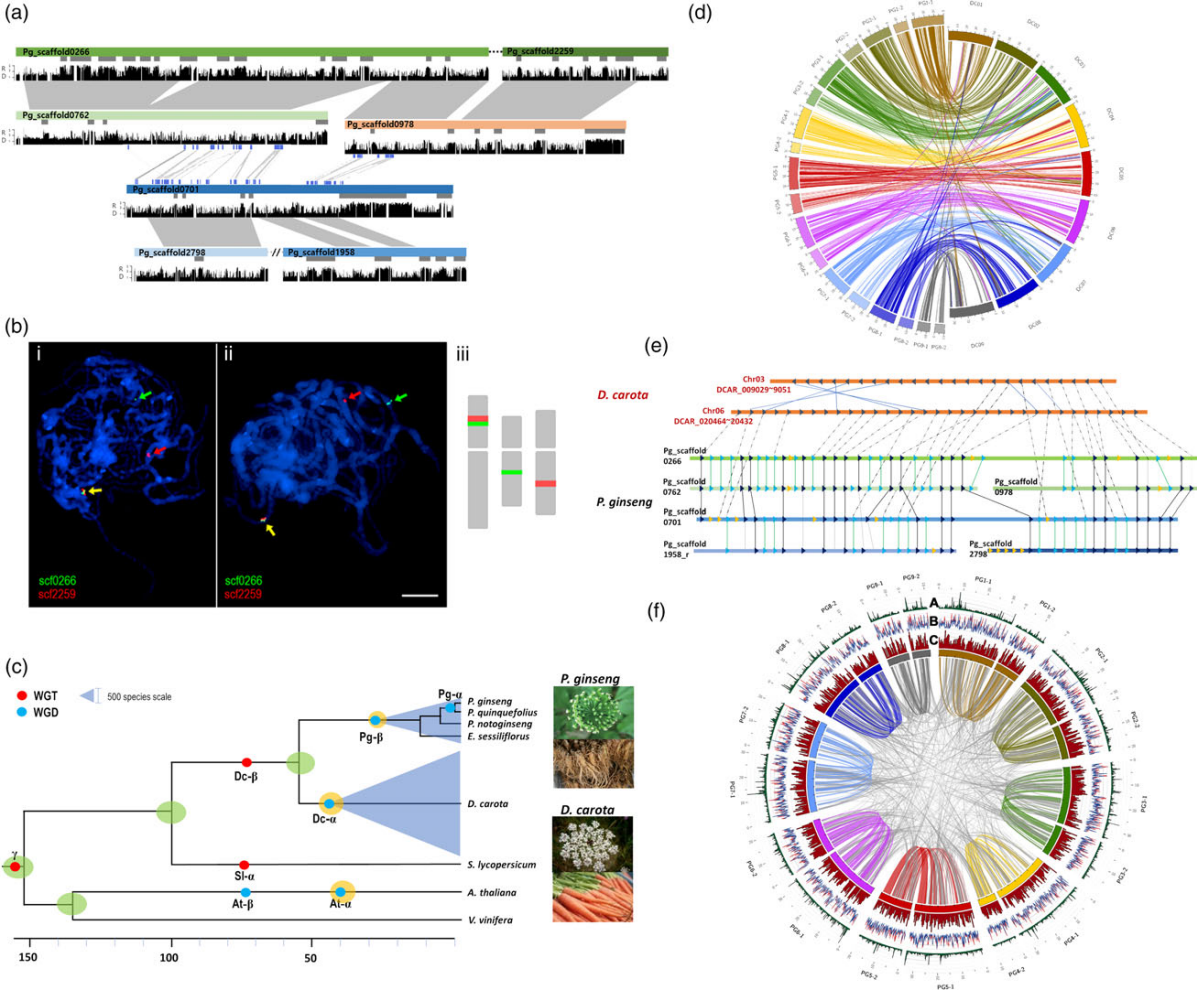
*Panax ginseng* genome structure and evolution. (a) Relationship between four paralogous blocks resulting from two WGD events. Block 1 composed of Pg_scaffold0266 and Pg_scaffold2259, Block 2 contained Pg_scaffold0762 and Pg_scaffold0978, Block 3 had just one scaffold, Pg_scaffold0701, and Block 4 comprised reverse of Pg_scaffold2798 and Pg_scaffold1958. (b) FISH analysis to confirm the chromosomal locations of scaffolds inferred to be adjacent by *zigzag* alignment from counterpart scaffolds. FISH probes designed to validate adjacent scaffolds, and applied to pachytene chromosomes indicated that Pg_scaffold0266 and Pg_scaffold2259 (i, ii) were adjacent but Pg_scaffold0762 and Pg_scaffold0978 were on different chromosomes (iii). (c) Evolutionary history of *P. ginseng*. The pale blue triangles signify species number in the Araliaceae (1500) and Apiaceae (3700). (d) Construction of 18 virtual superscaffolds based on *Daucus carota*. The artificial counterpart superscaffolds of *P. ginseng* were twice the number of the corresponding *D. carota* superscaffolds*,* because of Pg‐α WGD. (e) Syntenic analysis between *P. ginseng* and *D. carota*. The seven scaffolds described illustrated chromosomal rearrangements relative to two *D. carota* regions. (f) Circular map of 18 virtual superscaffolds of *P. ginseng* and distribution of SNPs with cv. YuP (A), repeats (B) and genes (C). Total identified repeats (red lines) and major LTR‐RT family, *PgDel* (blue lines).

Analysis of *P. ginseng* paralogs revealed two WGD events, at 2.2 Million Years Ago (MYA, called Pg‐α), and 28 MYA (Pg‐β), consistent with previous reports (Choi *et al*., 2013, 2014; Kim *et al*., 2017; Figure S4). Genes and flanking regions were highly collinear between paralogous Pg‐α WGD scaffolds (Figures 1a, S5 and S6), with 95% sequence similarity except in repeat‐mediated InDel regions (Figure 1a), whereas only genic regions have collinearity between Pg‐β WGD scaffolds. Utilizing Pg‐α WGD paralogs, a *zigzag* approach to identify putative contiguous counterpart scaffolds for ordering (Figure S5), established 453 recently duplicated collinear blocks. The collinear blocks consist of 1344 scaffolds covering 601.2 Mb with 29 953 genes. Our updated sequence scaffold (v0.8 to v1.0) and FISH analysis validated the *zigzag* approach for combining adjacent scaffolds, with some exceptions due to chromosomal rearrangement (Figures 1b and S7).

Gene sets of *P. ginseng* and four dicots (Arabidopsis, grape, tomato, carrot) were characterized by OrthoMCL (Li *et al*., 2003) and 1697 common orthologous gene clusters used to calculate *K*s value for clarifying major evolutionary events (Figure S4). Ginseng was estimated to have diverged from carrot (Apiaceae) ~51 MYA and Pg‐α and Pg‐β occurred independently from a WGD in the carrot lineage (Dc‐α) (Figure 1c). Manual ordering of *P. ginseng* scaffolds based on the carrot genome enabled us to construct 18 artificial counterpart superscaffolds (Figure 1d). Four paralogous ginseng blocks show collinearity with two carrot chromosomes (Figure 1e). The 18 ginseng superscaffolds showed the genomewide Pg‐α WGD and biased distribution of genes and repeats. More SNPs are also identified from gene‐rich regions (Figure 1f).

**Figure 2:**
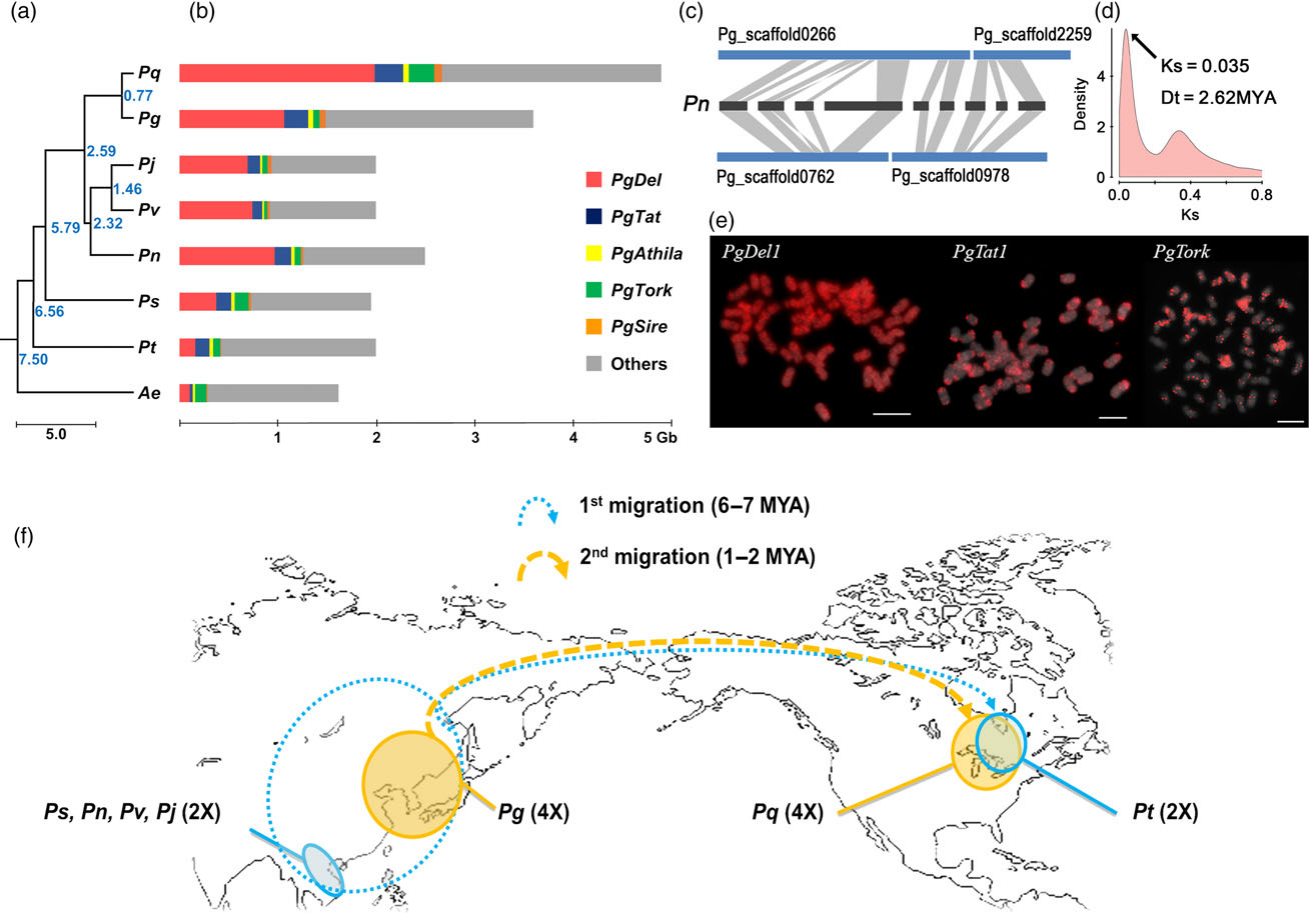
Evolutionary model for the *Panax* genus. (a) Phylogenetic tree based on complete chloroplast genome sequences. Estimated divergence times (MYA) are at the roots of branch extensions for *P. stipuleanatus* (Ps)*, P. notoginseng* (Pn)*, P. vietnamensis* (Pv)*, P. japonicas* (Pj)*, P. trifolius* (Pt)*, P. ginseng* (Pg)*, P. quinquefolius* (Pq) and *Aralia elata* (Ae), respectively. (b) Bar charts for genome size and estimated genome proportions of five major repeats. Estimated genome sizes depict the predicted amounts of *PgDel* (red)*, PgTat* (blue)*, PgAthila* (yellow)*, PgTork* (green) and *PgSire* (orange) LTR‐RT families (c) Homeologous scaffolds between *P. ginseng* and *P. notoginseng*. A total of nine *P. notoginseng* scaffolds matched two counterpart *P. ginseng* scaffolds. The listed *P. notoginseng* are scaffold11410, scaffold32646, scaffold10403, scaffold1534, scaffold27642, scaffold10246, scaffold1534, scaffold31849 and scaffold10465 in an order from left to right (d) *K*s distribution of orthologous genes between *P. ginseng* and *P. notoginseng*. The peak at 0.035 suggests 2.62 MYA divergence time between these species, similar to chloroplast genome‐based estimation (Figure 2a). (e) FISH analysis of Ty3/gypsy (PgDel1 and PgTat1) and Ty1/copia (PgTork) LTR retrotransposons to show their differential abundance. (f) Two rounds of intercontinental species migration. Solid lines indicate current habitats, whereas dotted lines indicate inferred past habitats and migrated vestiges. Blue lines indicate first migration of diploid *Panax,* and yellow lines indicate migration of tetraploid *Panax*.

**Figure 3:**
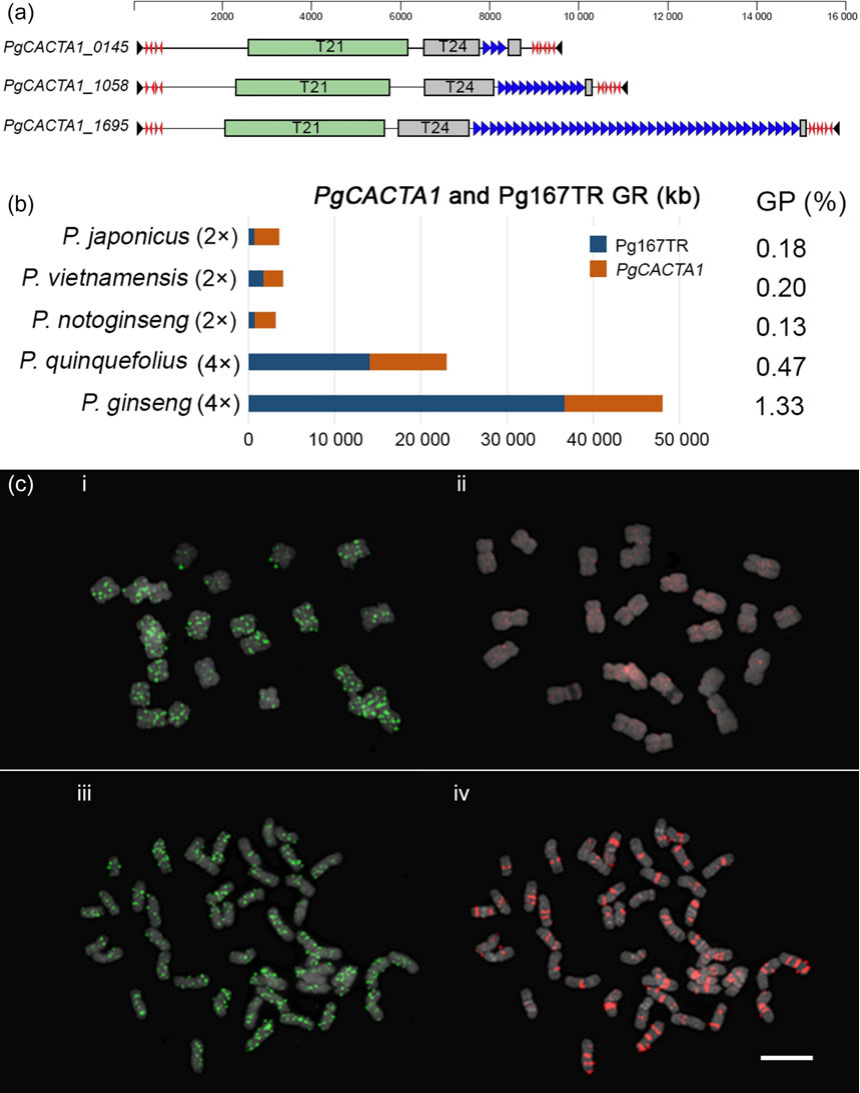
Characterization of *PgCACTA* harbouring extraordinary Pg167TR in *P. ginseng*. (a) Idiogram of three *PgCACTA* elements showing two transposase domains, transposase 21 (T21) and transposase 24 (T24), and different Pg167TR copy numbers (blue arrows). The Pg167TR sequences were often inserted into the last intron of T24. Subterminal repeats were longer at the 3’ end (red arrows), and 31‐bp TIRs were highly conserved. (b) Quantification of genomic Pg167TR within *Panax* species. Genome representation (GR) of Pg167TR showing the abundance in tetraploid *Panax* species. (c) FISH analysis with *PgCACTA* domain (i, iii) and Pg167TR (ii, iv) in *P. notoginseng* (i, ii) and *P. ginseng* (iii, iv). *PgCACTA* gene regions showed clear signals in both diploid and tetraploid species (i, iii), while Pg167TR showed very faint signals in *P. notoginseng* (ii) but showed highly abundant and distinct signals in *P. ginseng* (iv).

To understand evolution and speciation of *Panax* and its Araliaceae relatives, we obtained the complete chloroplast genomes and 45S nrDNA sequences from ten species as well as carrot. We included our previous data (Kim *et al*., 2015b, 2016a,b, 2017), and added newly generated chloroplast genomes and 45s nrDNA sequences from two more *Panax* species, (Table S14; Figure S8). Phylogenomic analysis of those 10 chloroplast genomes and 45S nrDNA genes indicated that the *Panax‐Aralia* lineages diverged ~7.50–7.97 MYA, following *Panax* speciation (Figures 2a and S9). Tetraploids *P. ginseng* and *P*. *quinquefolius* formed ~2.59 MYA (Figure 2a). Five uniquely enriched LTR‐RT families (*PgDel*,* PgTat*,* PgAthila*,* PgTork* and *PgSire*; Choi *et al*., 2014; Jang *et al*., 2017; Lee *et al*., 2017a) occupy >50% of the genome. *PgDel* LTR‐RTs largely account for genome size variation among seven *Panax* species (Figure 2b,e). *P. ginseng* has doubled orthologous sequences compared to diploid *P. notoginseng* (Zhang *et al*., 2017), and their modal *K*s value (0.035, Figure 2c,d) implies divergence 2.62 MYA, which is similar value to chloroplast analysis (Figure 2a). A unique high copy *En/Spm*‐like *CACTA* transposon (*PgCACTA1*) encoding two transposase genes and maintaining 31‐bp conserved terminal inverted repeats (TIR) has highly diverse copy number of Pg167TR (Waminal *et al*., 2016) in the last intron of the second transposase (Figures 3a and S10), providing a molecular barcode for identification of individual chromosomes (Figure S11; Waminal *et al*., 2016). The genome proportions of *PgCACTA1* and Pg167TR are diverse among *Panax* species and richer in tetraploids, especially *P. ginseng* (Figure 3b). Comparative FISH analysis with *P. notoginseng* using probes from *PgCACTA1* and Pg167TR transposase regions showed clear proliferation of Pg167TR in *P. ginseng* (Figure 3c) but little difference for the transposase regions between the two species.

### Ginsenoside biosynthesis

Ginsenosides, the major pharmacologically active compounds of ginseng, are triterpene saponins, of which more than 150 have been isolated from *Panax* plants (Christensen, 2009; Jia and Zhao, 2009). To characterize ginsenoside biosynthetic machinery and metabolic utilization, a genome‐scale metabolic network was newly constructed based on established procedures (Thiele and Palsson, 2010), covering 4946 genes catalysing 2194 reactions and 2003 unique metabolites (Data S1) with a global overview in Figure S12.

**Figure 4:**
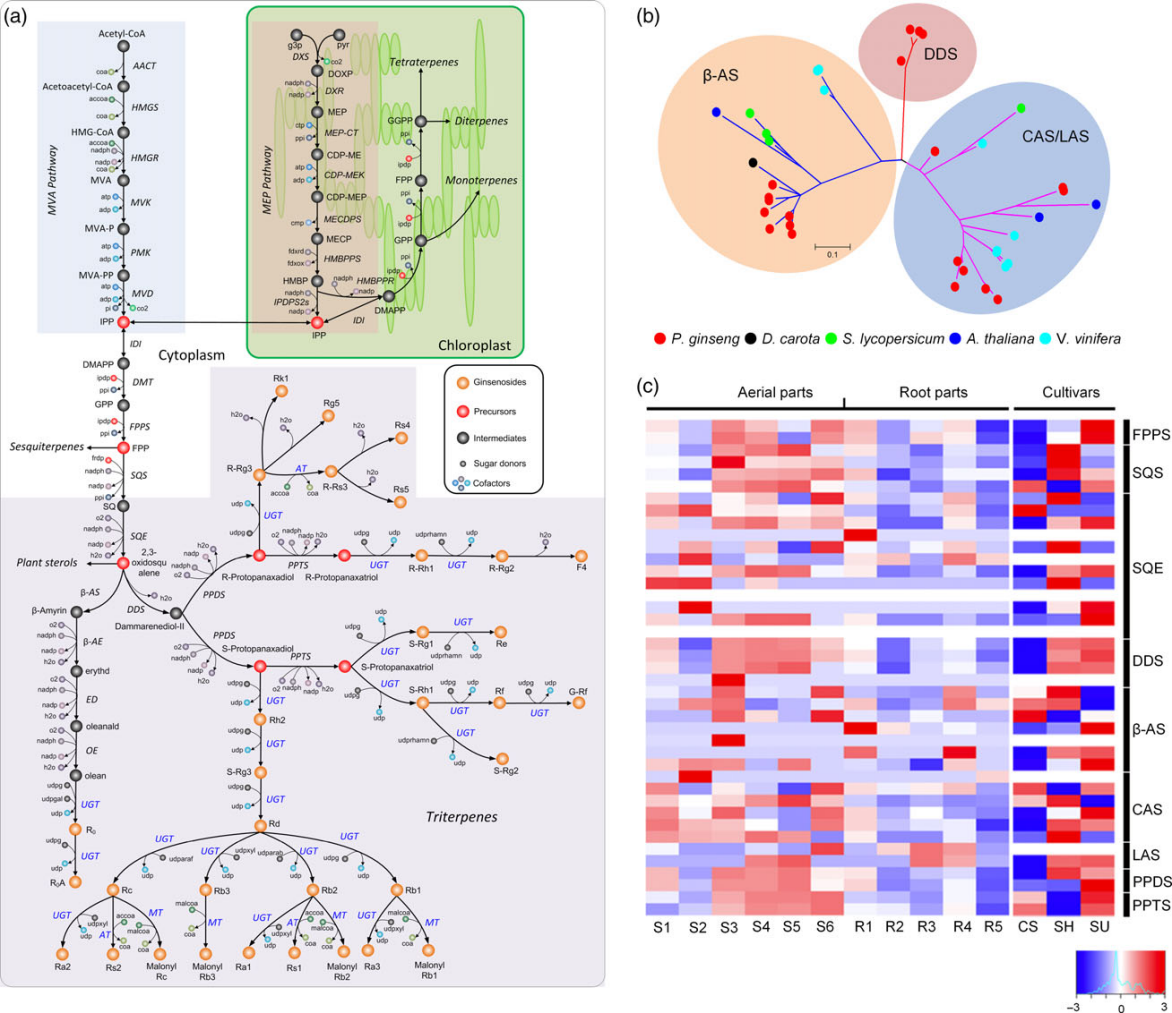
Ginsenoside biosynthesis model and related genes in *P. ginseng*. (a) Overview of the ginsenoside biosynthetic pathway in *P. ginseng*. The blue coloured uridine 5′‐diphospho‐glucuronosyltransferases (UDP‐glucuronosyltransferase, UGTs) are unknown enzymes involved in the glycosylation of ginsenosides. Reaction and metabolite abbreviations can be found in Data S1. (b) A phylogenetic tree of oxidosqualene cyclases (OSCs) in *P. ginseng*. OSC genes, including dammarenediol synthase (DDS), β‐amyrin synthase (β‐AS), lanosterol synthase (LSS) and cycloartenol synthase (CAS), were identified from *P. ginseng* (red), *D. carota* (black), *S. lycopersicum* (green), *A. thaliana* (blue) and *V. vinifera* (cyan) by KEGG and BLASTP searches. (c) Heatmap shows TMM normalized expression values of putative downstream genes involved in ginsenosides biosynthesis. Expression in above‐ground tissue (S1: immature fruit, S2: mature fruit, S3: flower, S4: 1‐year‐old leaves, S5: 5‐year‐old leaves, S6: 6‐year‐old stem) and subterranean parts (R1: 1‐year‐old main body roots, R2: 6‐year‐old main body roots, R3: 6‐year‐old lateral roots, R4: 6‐year‐old rhizomes, R5: 6‐year‐old dormant roots) are depicted. Similarly, expression of downstream genes is shown between adventitious roots of *P. ginseng* cultivars, CS, SH and SU.

Ginsenosides are biosynthesized through cyclization, hydroxylation and glycosylation of 2,3‐oxidosqualene that is synthesized via mevalonate (MVA) and 2‐C‐methyl‐D‐erythritol‐4‐phosphate (MEP) pathways. In most plants, 2,3‐oxidosqualene is subsequently cyclized into cycloartenol, α‐, β‐amyrin or lupeol, to be further converted to phytosterols and pentacyclic triterpenoids (Benveniste, 2004). In *P. ginseng*, an additional cyclic compound, dammaranediol, can be biosynthesized by a specific cyclase then oxidized through a set of cytochrome P450 enzymes to form the major dammarane‐type sapogenins [protopanaxadiol (PPD)/protopanaxatriol (PPT)], while the minor oleanane‐type aglycone (oleanolic acid) is biosynthesized from β‐amyrin. These precursors are further glycosylated via several UDP‐glycosyltransferases (UGTs) to synthesize various types of ginsenosides (Figure 4a). Twelve squalene epoxidase (SQE) genes were identified in *P. ginseng*, twice as many as in other plants (Table S15), suggesting increased ginsenoside precursor production. Twenty *P. ginseng* oxidosqualene cyclase (OSC) genes were found in the biosynthesis of dammarane‐/oleanane‐type ginsenosides [dammarenediol synthase (DDS), β‐amyrin synthase (β‐AS)] and sterols (lanosterol synthase (LSS), cycloartenol synthase (CAS)].

Phylogenetic analysis of OSC families found DDS to be specific to *P. ginseng* (Figure 4b), suggesting that DDS and production of dammarane‐type ginsenosides originated in *Panax*. Of 383 *P. ginseng* cytochrome P450 genes, two candidate protopanaxadiol synthase (PPDS) and two protopanaxatriol synthase (PPTS) genes were identified by homology search against curated PPDS (Han *et al*., 2011) and PPTS, respectively. In the last glycosylation step, 226 UGTs were annotated and eleven identified as candidate UGTs associated with elevated expression pattern upon methyl jasmonic acid (MeJA) treatment (Figure S13), which is well‐known elicitor for inducing secondary metabolites (Han *et al*., 2011, 2012). These candidate UGTs could be involved in synthesis of PPD‐type ginsenosides, as MeJA triggers mainly PPD‐type (Oh *et al*., 2014).

The high ginsenoside contents for which older (above 4–6 years) *P. ginseng* roots are harvested might reflect transportation from shoot tissues rather than active biosynthesis. Downstream genes (SQE, DDS, PPDS and PPTS) in the ginsenoside biosynthetic pathway showed higher expression in leaves (1 year old and 5 years old) than roots (1‐ and 6‐year‐old main body roots, lateral roots and rhizomes; Figure 4c). Co‐expression analysis across RNA‐Seq samples from ChP showed that three highly expressed DDS genes among 20 OSC are co‐regulated with several SQE genes, and disrupting function of either DDS or SQE affects *P. ginseng* ginsenoside production (Han *et al*., 2010; Tansakul *et al*., 2006). This implies that DDS and SQE may be important enzymes with which ginsenoside production co‐evolved. Indeed, higher expression of downstream genes in *P. ginseng* cultivars Cheongsun (CS) and Sunhyang (SH) than Sunun (SU; Figure 4c) is associated with higher ginsenoside content (Table S16). While many CPY450 and UGTs are not yet characterized with respect to different types of ginsenosides (Figure 4a), dynamic changes in expression of various genes were observed across the metabolic network (Figure S14), providing a foundation for *in silico* analysis and ultimately empirical metabolic engineering.

### Gene families responsible for environmental adaptation

Differentially expressed genes (DEGs) were identified with two/three biological replicates abiotic stress‐treated RNA‐Seq samples. In detail, 703, 152 and 23 genes were shown different expression in response to drought, cold and salt, respectively (Figure S15). DEG analysis was also performed between non‐heat‐treated leaves and heat‐treated (1 and 3 weeks) leaves of three replicates. In total, 1409 genes were identified as DEG after 1 and 3 weeks of heat treatment (Figure S15). Altogether, 1880 genes were found to be differentially expressed (DE) and the numbers of DE genes including up‐ or down‐regulated genes are represented in Figure S15. Majorly, fatty acid desaturase (FAD) and light‐harvesting chlorophyll a/b binding (CAB) proteins were highly responsive to abiotic stresses including drought, salt, cold and heat. An unprecedented 85 FAD genes were found in *P. ginseng*, almost three times as many as in model annual plants (Table S17). Phylogenetic analysis revealed diverged FAD gene structures to include a *Panax*‐specific subgroup (acetylenic FADs), and a carrot‐ and *Panax*‐specific FAD‐like subgroup (Figure S16).

**Figure 5:**
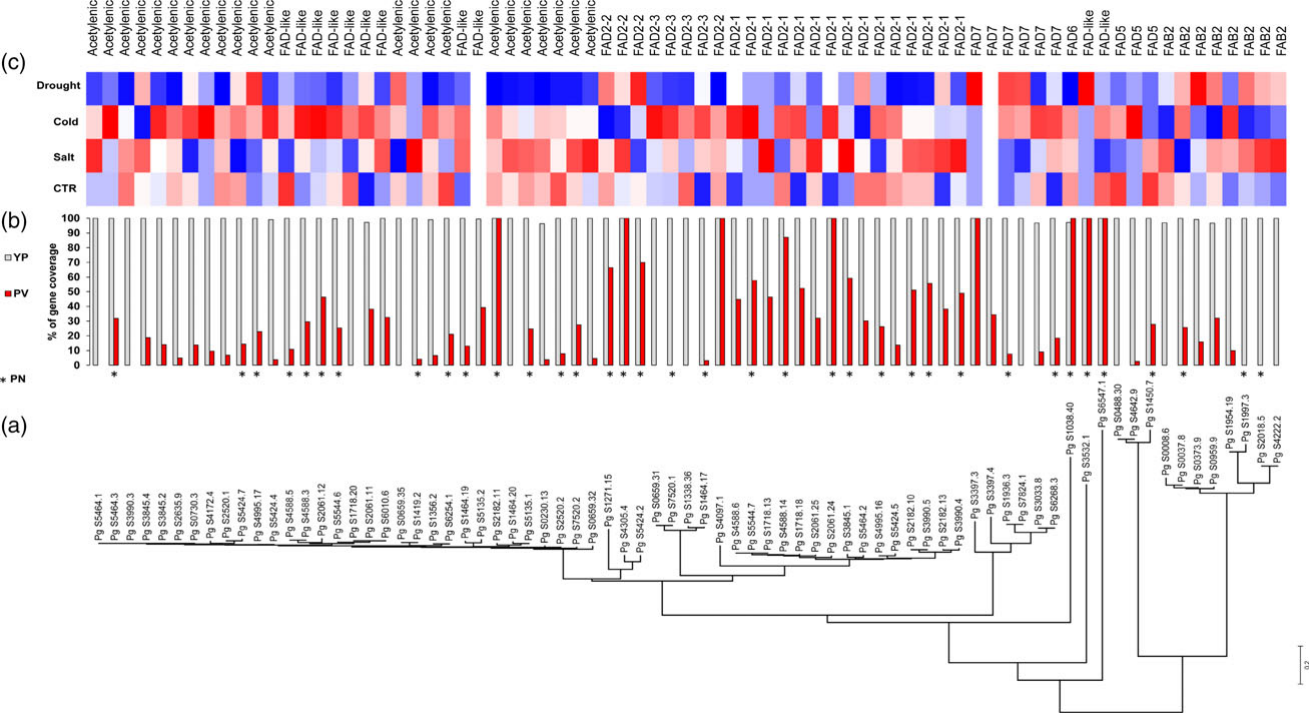
Classification and expression of FAD genes. (a) Phylogenetic analysis of FAD genes. (b) Mapping coverage for coding (CDS) genes using 10× coverage WGS reads from tetraploid *P. ginseng* cv. YuP (white bars) and diploid *P. vietnamensis* (red bars). Orthologous FADs in diploid *P. notoginseng* (PN) denoted as * under the bar graphs. (c) TMM normalized expression of each gene under control (CTR), drought, salt and cold stress conditions. The FAD subclass is represented at the top to show expansion of specific subclasses and its role in abiotic stress responses.

Many FAD orthologs (Figure 5a) in tetraploid *P. ginseng* cv. Yunpoong (YuP) were not found in diploids *P. vietnamensis* (Figure 5b) and *P. notoginseng*, with only thirty‐six *P. notoginseng* genes having orthologous relationships to *P. ginseng* FADs. The newly evolved *P. ginseng* FADs showed higher expression in cold stress (Figure 5c), suggesting a role in cell membrane fluidity contributing to freezing tolerance.

An unprecedented 49 CAB genes were found in *P. ginseng*, with family expansion due to retention of whole genome duplicated copies Figure S17). All 49 CAB genes showed expression, albeit in various tissues, with significantly increased expression in leaves and decreased expression during abiotic stresses, especially drought and heat (Figure S18). All CAB orthologs were found in both *P. ginseng* and *P. vietnamensis* (Figure S19). The expansion of *Panax* CABs is consistent with shade adaptation, enabling efficient photosynthesis in low light. Some TF families showed *P. ginseng*‐specific expansion, notably, FAR1 (far‐red‐impaired response), HRT (*Hordeum* repressor transcription) and CSD (cold‐shock domain) families (Figure S20; Table S11), indicating that expanded regulatory capacity also contributed to shade and cold adaptation.

## Discussion

The genome sequence of *P. ginseng* opens a route to functional and molecular breeding of economically important herbaceous perennials within the Araliaceae family. The genome sequence covers ~80% of the estimated genome size (~3.6 Gbp) and identified two rounds of WGDs unique in the Araliaceae family. The recent, 2.2 MYA, WGD event (Pg‐α) contributed substantially to duplicated genes and genome structure of *P. ginseng*, with gene number about twice that of diploid *P. notoginseng* and other diploid plants. Following this recent WGD, 99% and 95% of homology showed between paralogous genes and its flanking regions, respectively, except TE‐mediated sites complicating genomic analysis in *P. ginseng*. Like other plants, LTR‐RTs were most abundant in ginseng genome in which LTR/Gypsy accounted for 49%, especially one *PgDel* family extremely abundant occupying 30% of whole genome sequence. Cytogenetic mapping of major *P. ginseng* TEs revealed hybridization of different repeat families to different chromosomal niches (Figure S21). *PgDel1* hybridized to the entire chromosomes, supporting their predominant abundance in the ginseng genome (Choi *et al*., 2014).

Insertion time estimation using LTR sequence of intact major LTR‐RTs indicated that most of LTR‐RTs were expanded recently after Pg‐α WGD in *P. ginseng* (Figure S22). We also assumed that one more expansion of major LTR‐RTs occurred around 5–6 MYA, according to repeat GP of *P. stipuleanatus* and *P. trifolius* being half of the others diploid *Panax* species (Figure 2a,b). Although Class II TEs generally have lower genome proportion (GP) than Class I, they are known to be important gene regulators in a genome (Gao *et al*., 2016). We identified a novel *En/Spm* (CACTA) element *PgCACTA1* in the ginseng genome and its insertion at high AT regions and conservation of the TIR sequences with other *PgCACTA* elements indicate its relatively recent insertion. Comparative analysis of *PgCACTA* abundance among *Panax* species showed preferential expansion of the Pg167TR in tetraploids, particularly in *P. ginseng*, whose genome contains 1.3% of *PgCACTA* (Figure 3b). Comparative FISH data between *P. ginseng* and a diploid relative, *P. notoginseng*, supported the expansion of Pg167TR in *P. ginseng* (Figure 3c). The amplification pattern of *PgCACTA* and Pg167TR suggests a tetraploid lineage‐specific evolutionary pathway associated with the recent Pg‐α WGD. These data imply that during the Pg‐α WGD, *PgCACTA* amplification could have been triggered in response to genomic shock as in other plants (Fedoroff and Bennetzen, 2013; Kalendar *et al*., 2000). Concomitant to this amplification was the amplification of Pg167TR, which led to the distinct chromosomal loci in tetraploid ginseng.

Further, we postulate that major evolution events in *Panax* species including two rounds of WGD and intercontinental species migrations were related to recurrent glaciations (ice ages) and global warming. The estimated 51 MYA Araliaceae–Apiaceae divergence falls early in the Eocene (56–34 MYA) global warming (Figure 1c), with the 1500 Araliaceae species (Gao *et al*., 2013) proliferating following Pg‐β WGD 28 MYA. Complete chloroplast genome‐based molecular clocks suggest the history of recent divergence of *Panax* species. The common diploid *Panax* ancestor, which was a heat‐susceptible shade‐loving plant, was distributed over the Qinghai–Tibetan Plateau by divergence with the Aralia genus ~7.5 MYA (Li and Wen, 2016). *Panax trifolius* is the unique *Panax* diploid in North America and was estimated to diverge ~6.6 MYA, prior to divergence of the other four *Panax* diploid species in Asia, suggesting that diploid *Panax* species proliferated to North Asia and crossed into North America during that period (Figure 2f). Pliocene (5.33–2.58 MYA) speciation of diploid *Panax* was associated with global warming, while Pleistocene (~2.58 MYA) glaciation was associated with their extinction. Allotetraploidization between these diploids occurred sequentially, and an allotetraploid ancestor of the current *P. ginseng* may have survived in Northeast Asia by gaining overwintering ability. Cold‐susceptible *Panax* diploids may have been isolated at high altitude in warm Southern Asia, favouring speciation (heat‐island effect), with ~10 extant diploid *Panax* species at risk of extinction from global warming. The ancestor of *P. quinquefolius* may have migrated to North America ~1.2–0.8 MYA in a glacial period (Figure 2f; Choi *et al*., 2013).

For a better understanding of the metabolic paradigm in *P. ginseng*, a genome‐scale metabolic network was reconstructed in this study, which leads to *in silico* metabolic engineering that could predict candidate genes associated with overproduction of desired metabolites and thus accelerate overall metabolic engineering process. Only plants in the genus *Panax* actively biosynthesize various types of ginsenosides (Kim *et al*., 2015d), which was explained by the taxonomic specific origin of DDS genes. We have also demonstrated the candidate genes including DDS and SQE controlling the accumulation of ginsenosides with transcriptome and metabolome data. These results provide essential targets to increase the production of ginsenosides through latest biotechnological approaches.

The recent allotetraploidization event (Pg‐α) might have promoted environmental adaptation such as survival of freezing temperatures. A well‐characterized phenomenon demonstrates that temperature modulates membrane fluidity, which is the major site of freezing injury (Shewfelt, 1992; Thomashow, 1999). It is also known that the role of FADs in cold acclimation in various plant species (Khodakovskaya *et al*., 2006; Román *et al*., 2012; Thomashow, 1998). In addition, the divergent FAD genes have been associated with synthesis of divergent fatty acid structures that play major role against biotic/abiotic stresses (Cao *et al*., 2013). As compared to diploid ginseng, the polyploid ginseng species such as *P. ginseng* and *P. quinquefolius* have been commonly found in the habitat of Northeast Asia and North America, respectively, where freezing temperature prevails in the winter. Therefore, the expansion of FAD genes with diverse FAD structures in *P. ginseng* or polyploidization of ginseng species might have led to freezing tolerance.

Light is a limiting factor for the ginseng cultivation and plays role in ginsenoside production. Ginseng has been grown under canopy or artificial shade; however, the reason behind this process is largely unexplored. It is obvious that the ginseng plant should have acquired a novel mechanism to ensure an efficient photosynthesis under low‐light conditions. The light‐harvesting chlorophyll a/b binding proteins (LHCPs or CAB) are the key components of the photosynthesis antennae complexes, which transfer the light energy to the reaction centres of photosystem I (PS I) and photosystem II (PS II) where the light energy is converted to form chemical bond energy (i.e. NADPH and ATP). Intriguingly, *P. ginseng* genome contains more CAB genes than any plant species to date, which was supported by RNA‐seq expression (Figure S18). Equivalently, a total of 53 genes (including pseudogenes) were also identified in the genome of brown algae (*Ectocarpus siliculosus*; Cock *et al*., 2010) and that expansion was attributed to adapt to variable or dim light conditions. We have also deduced the ability of ginseng plants to cope with low‐light environments is related to its as‐yet‐unprecedented expansion in number of CAB genes, with decreased expression during drought and heat stresses. Intriguingly, estimation of presence/absence of orthologous gene copies in *P. vietnamensis* revealed the abundance of CAB genes in both shade plants, tetraploid and diploid ginseng species (Figure S19).

## Conclusion

The genome sequence clarifies the evolution, shade adaptation, and medicinal properties of *P. ginseng*. Two Araliaceae‐specific WGDs played key roles in environmental (shade and freezing) adaptation and medicinal importance (dammarane‐type ginsenoside production), the former also providing information that might apply to improvement of other cultigens. The widespread importance of collecting and cataloguing crop relatives is especially urgent in *Panax*, in which extant diploid relatives are at risk of extinction from global warming, progenitors of cultivated tetraploids are already extinct, and wild tetraploids are endangered by over‐harvesting (Baeg and So, 2013; Court, 2000).

## Methods

### *De novo* sequencing, assembly and quality evaluation

DNA from leaves of 4‐year‐old ChP, an elite Korean cultivar, was used for sequencing and assembly. The ChP was cultivated in a ginseng experimental field of research farm (College of Agriculture and Life Science, Seoul National University, Suwon, Korea) and used for isolation of genomic DNA and total RNA. To reduce heterogeneity, we used DNA from three individuals. Whole genome shotgun reads of ChP were generated using Illumina platform (HiSeq2000 and MiSeq) at National Instrumentation Center for Environmental Management (NICEM), Macrogen Co. (Seoul, Korea), and LabGenomics Co. (Seongnam, Korea). The five paired‐end (PE) libraries (with 200–600 bp insert sizes) were sequenced into 746 Gbp for primary assembly, and the 365 Gbp was sequenced from four mate‐pair libraries with 1.5 kb, 3 kb, 5 kb and 10 kb insert for scaffolding. First, low‐quality reads and duplicated reads were eliminated using SOAPfilter 2.0 of SOAPdenovo package (Luo *et al*., 2012) with default parameter. Furthermore, low‐frequency reads were eliminated based on *k*‐mer frequency by SOAPec 2.0 with KmerFreq_HA 2.0 and Corrector_HA 2.0, which cannot support for initial contig assembly. Genome size estimation was conducted by flow cytometry and 23 bp *k*‐mer frequency analysis with JELLYFISH (Marçais and Kingsford, 2011). Taken together, the genome size of *P. ginseng* was estimated to range between 3.3 and 3.6. The *k*‐mer frequency‐based genome size, 3.6 Gbp, was used for further analysis and discussion for the genome composition. The genome assembly was mainly conducted using SOAPdenovo2. The contigs containing length over 1 kb and filtered mate‐pair reads were used for scaffolding with SSPACE followed by error correction by in‐house Perl scripts (Phyzen, Seongnam, Korea).

### Validation of genome assembly

The assembled draft sequence was validated by mapping of MP reads and alignment with reported bacterial artificial chromosome (BAC) sequences (Choi *et al*., 2014; Jang *et al*., 2017). First, the 1382 million (M) of 1385 M filtered MP reads were mapped to assembled sequence through BWA (Li and Durbin, 2009) (v0.7.12) with default parameter, of which 536 M reads were mapped with paired ends. The assembled genome sequences were compared to 13 BACs composed of 15 contigs, which were sequenced using PacBio RSII platform and ABI3730 sequencer. Each scaffold matched with BAC clones sequence was identified through BLAST analysis and visualized with dotplot by PipMaker (Schwartz *et al*., 2000). Furthermore, the genome assembly completeness was validated using Benchmarking Universal Single‐Copy Orthologs (BUSCO_v2; Simão *et al*., 2015).

### Transcriptome sequencing and analysis

Tissues and cultivars used in this study were described in Table S7. Dormant roots with healthy rhizomes of 1‐year‐old cv. ChP plants were obtained from the Ginseng Research Division, National Institution of Horticultural and Herbal Science, Rural Development Administration (Eumseong, Korea). After storage for more than 1 month at 4°C to break dormancy breaking, the roots were planted in soil and grown for 4 weeks to become plants with fully expanded leaves under normal growth condition (24°C, relative humidity 60%, and continuous light of 40 μE/m^2^/s). These plants grown for 4 weeks were sampled as controls, immediately before stress treatment. For cold treatment, the plants were held at 4°C for 24 h (with relative humidity and light conditions the same as the normal growth condition). For salt treatment, pots with plants were submerged in 100 mm NaCl solution for 24 h to treat only root parts with salt stress (temperature, relative humidity and light condition were the same as the normal growth condition). For drought stress, plants were removed from soil and air‐dried on 3MM paper for 24 h under the normal growth condition. For heat treatment, the plants were treated with 30 (±1)°C for 1 week and 3 weeks (relative humidity and light conditions were the same as the normal growth conditions). After stress treatment, whole plant (leaves, stems and roots) were sampled, immediately frozen using LN_2_, and stored at −70°C before total RNA isolation.

Total RNAs from each sample were extracted using RNeasy Plant kits (QIAGEN, Hilden, Germany) and/or Hybrid‐R kits (GeneAll, Seoul, Korea) according to the manufacturer's instructions, and used for construction of 300‐bp PE libraries using an Illumina TruSeq RNA sample preparation kit according to the manufacturer's instructions. These libraries were pooled and sequenced by Illumina HiSeq2000 and NextSeq500 platforms (Table S7). The resulting RNA‐Seq reads were mapped to the *P. ginseng* draft genome and assembled using HISAT (Kim *et al*., 2015a) and StringTie (Pertea *et al*., 2015), respectively. *De novo* assembly was performed using Trinity (Grabherr *et al*., 2011) to obtain full‐length transcripts. All RNA‐Seq samples were normalized using Trimmed Mean of M values (Dillies *et al*., 2013) (TMM). Analysis of differential gene expression was performed using edgeR (Robinson *et al*., 2010) with false discovery rate (FDR)‐adjusted *P*‐value of 0.01. Transcriptomes of 22 ChP samples including normal tissues and abiotic stress‐treated samples were also analysed using 26 SMRT cells with P6‐C4 chemistry of the PacBio RSII platform. Generated sequences were classified and clustered by the PacBio Iso‐Seq analysis procedure (ver. 0.1) with default parameters ( http://www.pacb.com) to generate high‐quality (HQ) consensus isoform sequences (99% consensus accuracy based on Quiver). The HQ sequences were further processed to remove PCR chimeras and redundant sequences by cd‐hit‐est (Li and Godzik, 2006), and final HQ nonredundant (nr) isoform sequences were obtained based on genome positional coordinates.

### Genome annotation

The IPGA pipeline was used for genome annotation, incorporating evidence from protein and RNA‐Seq mapping and *ab initio* gene prediction to determine consensus gene models by EVM (Haas *et al*., 2008), that were curated using PacBio transcript sequences. The alternative splicing transcripts for the final curated protein‐coding genes were identified using reference‐based assembly generated by PacBio and Illumina sequencing data. Then, the reference‐guided transcripts and annotated protein‐coding genes were compared to identify novel isoforms using cufflink utility. Further, those novel isoforms were used to find the specific splicing events (i.e. skipping exon, mutually exclusive exons, alternative 5′ or 3′ splice site, retained intron and alternative first and last exon) using SUPPA (Alamancos *et al*., 2015). LncRNAs were identified from the reference‐guided transcriptome assembly. From the total transcripts, transcripts with ORF ≥ 100 amino acids and length ≤200 nucleotides, having homology hit to the swiss‐prot protein database, Pfam domains and other types of noncoding RNAs (tRNA, rRNA, snRNA, snoRNA), were discarded. Further, transcripts that span over 40% to repeat‐masked genomic region and contained partially at protein‐coding genes (IPGA v1.1) were discarded. Finally, the coding potential was accessed for the remaining transcripts using CPC with score ≤−1.0 and CPAT with score <0.39. A total of 19 495 lncRNA transcripts were identified using the above criteria. Transcription factor genes in the *P. ginseng* genome were identified and compared with corresponding genes in other plant genomes using iTAK 1.6b standalone (Zheng *et al*., 2016) with default parameters. A *P. ginseng* small RNA library generated by Mathiyalagan *et al*. (2013) was used for conserved miRNA prediction using mireap v0.2 ( https://sourceforge.net/projects/mireap/). The predicted miRNAs with match to miRBase (v21) ( http://www.mirbase.org/) were referred to as conserved miRNAs. The target prediction was performed for conserved miRNAs using psRNATarget (Dai and Zhao, 2011). Functional descriptions were assigned to annotated genes using BLASTP search (*E*‐value: 1E‐05) to NCBI Nr, Arabidopsis, tomato and INTERPRO protein databases (Zdobnov and Apweiler, 2001). GO enrichment analysis was performed using Fisher's exact test with multiple testing correction of FDR with cut‐off 0.05. The *P. ginseng* repeat library was constructed from eight reported transposable elements and consensus repeats characterized with pre‐identified LTR‐RTs (Choi *et al*., 2014; Jang *et al*., 2017) and RepeatModeler (Smit and Hubley, 2010), and genomewide repeat content of the assembled genome was calculated with RepeatMasker (Smit *et al*., 1996).

### Resequencing for genetic diversity

For investigation of genetic diversity in *P. ginseng*, one cultivar Yunpoong (YuP) was selected and resequenced, which is one of the oldest cultivars in Korea and morphologically different with ChP. DNA extraction and library construction with 101 bp were conducted under same condition as ChP. The sequencing results consisted of 486 M reads covered 49 Gbp (13× coverage), of which 46 Gbp of sequence were remained after filtering process. The mapping of filtered reads was conducted by default parameter of BWA (v0.7.12) using mem option. The Picard tools (v1.136) were used for conversion of Bam file to Sam file and remove PCR duplicates. The variation calling was conducted by GATK under RealignerTargetCreator, IndelRealigner and UnifiedGenotyper. The final variations were counted to 402 980 under following filtering criteria, 5–200 of depth coverage and 0.9 of variation allele frequency. Other *Panax‐*related species were also sequenced for comparative analysis with *P. ginseng*, especially for repetitive DNA in each genome. All sequences uploaded in the National Agricultural Biotechnology Information Center (NABIC, http://www.nabic.rda.go.kr) database under accession numbers of NN‐0189‐000001 (*P. quinquefolius*), NN‐1913‐000001 (*P. notoginseng*), NN‐1914‐000001 (*P. japonicus*), NN‐1915‐000001 (*P. vietnamensis*), NN‐0665‐00001 (*P. trifolius*)*,* NN‐0666‐000001 (*P. stipuleanatus*), NN‐0919‐000001 (*Aralia elata*), NN‐1907‐000001 (*Eleutherococcus gracilistylus*), NN‐1893‐000001 (*Kalopanax septemlobus*) and NN‐0168‐000001 (*Eleutherococcus senticosus*).

### Genome evolution

A reciprocal all‐vs.‐all BLAST hit approach was used to identify homologous *P. ginseng* genes, which were clustered using an in‐house Python script. Synonymous substitution (*Ks*) was calculated for duplicated genes using codeml in PAML package (Yang, 2007). Paralogous and syntenic collinear blocks were characterized using MCScanX (Wang *et al*., 2012). Sequence comparison was conducted using BLASTZ (Schwartz *et al*., 2003), PipMaker (Schwartz *et al*., 2000) and SynMap (Lyons *et al*., 2008). A *zigzag* approach was designed that assigned the adjacent scaffolds based on the counterpart paralogous scaffold information. Collinear blocks consisted of several scaffolds which sharing more than five paralogous genes with *Ks* values <0.2. Gene sets from Arabidopsis, grape, tomato and carrot were used for ginseng orthologous gene family clustering by OrthoMCL (Li *et al*., 2003). The complete chloroplast genomes and 45S nrDNA were newly assembled for *P. trifolius* and *P. stipuleanatus* based on dnaLCW method (Kim *et al*., 2015c). Phylogenetic trees of the chloroplast genome and 45S nrDNA of *Panax*‐related species were generated by Bayesian Inference using BEAST (version 1.8.1) (Drummond and Rambaut, 2007), and divergence time was calculated using the root age between *P. ginseng* and *D. carota*. The collinear region between *P. ginseng* and *D. carota* was characterized using MCScanX. The number of identified collinear *P. ginseng* scaffolds was 970 that harboured 25 091 genes and occupied 476 692 022 bp, of which 13 356 genes had collinear orthologous relationship with 10 381 of *D. carota* genes. Manual ordering of *P. ginseng* scaffolds based on gene order of *D. carota* chromosomes and constructed 18 artificial superscaffolds of *P. ginseng*. The genomic proportion of the major repeats was calculated as the total amounts of nucleotides mapped to the major repeats divided by the sum of all nucleotides in each data set (GP (%) = (masked read length/total read length) × 100). Repeat amount was used as an indicator of the actual amounts of repeats in a total genome which calculated as repeat amount = (masked read length × total read length) × (actual genome size/amount of WGS data used).

### Genome‐scale metabolic network reconstruction

A *P. ginseng*‐specific metabolic network was assembled using information from KEGG (Kanehisa *et al*., 2012) and BioCyc (Caspi *et al*., 2014) databases based on sequence homology of *P. ginseng* with tomato, rice and Arabidopsis. The consensus network was then curated by removing duplicated reactions and verifying elemental balances using the COBRA toolbox (Schellenberger *et al*., 2011). Thermodynamic reversibility of the reactions was assessed using databases such as MetaCyc and BRENDA (Chang *et al*., 2014). Isoenzyme and subunit information for reactions were added to the network based on gene annotations. Secondary metabolic pathways of *P. ginseng* including the ginsenosides were added based on the combined evidence from gene annotations and literature‐based biochemical information. Enzyme compartmentalization of the *P. ginseng* network was inferred from information available in a rice genome‐scale metabolic network (Lakshmanan *et al*., 2015) and Plant‐mPLoc (Chou and Shen, 2010). Dead‐ends in the network were filled by the GapFind algorithm (Kumar *et al*., 2007) in the COBRA toolbox.

### Identification of genes in ginsenoside biosynthetic pathway

Genes involved in ginsenoside biosynthesis were identified using KEGG annotation and BLASTP against reference enzyme genes retrieved from KEGG and MetaCyc databases ( http://metacyc.org/) with *E*‐value cut‐off of 1E‐05. The key candidate genes were identified by co‐expression analysis across RNA‐Seq samples from ChP with Pearson correlation coefficients (PCC). MeJA treated RNA‐seq data sets in *P. ginseng* cv. CS were used from Lee *et al*. (2017b).

### Identification of FAD and CAB genes

Fatty acid desaturase genes in *P. ginseng* were identified using Pfam accessions PF00487, PF11960 and PF03405 from an INTERPRO scan. InterPro analysis was also used to identify FADs in other selected plant species. CAB genes in *P. ginseng* and other species were identified using Pfam domain PF00504 from Interpro annotation. Phylogenetic trees were generated by MEGA 6.0 (Tamura *et al*., 2013).

### Estimation of orthologous gene copies using low‐coverage WGS

The low‐coverage (~10×) WGS data from *P. ginseng* cv. YuP (tetraploid) and *P. vietnamensis* (diploid) were utilized for estimation of presence/absence for the orthologous gene copies. The paired‐end reads were quality‐trimmed and pooled together as single reads. Based on OrthoMCL, the orthologs genes were selected from gene families such as FAD and CAB. The pooled reads of *P. ginseng* cv. YuP and *P. vietnamensis* were mapped to the orthologs genes (only CDS region) separately using BWA. Then, the percentage of each gene coding length (bp) covered by mapping reads was determined to check whether the coding region of a gene present in the whole genome sequencing library of each species.

### Fluorescence *in situ* hybridization (FISH)

Root mitotic chromosomes were prepared using the method previously described (Waminal *et al*., 2012). The retrotransposon probes were labelled directly or indirectly through nick translation of PCR product for each retrotransposon subfamilies. For direct labelling, either Texas Red‐5‐dUTP (NEL417001EA, Perkin Elmer, Waltham, USA) or Alexa Fluor 488‐5‐dUTP (C11397, Invitrogen, Eugene, USA) was directly incorporated into the DNA probes. For indirect labelling, either digoxigenin‐11‐dUTP (11‐745‐816‐910, Roche, Mannheim, Germany) or biotin (11‐745‐824‐910, Roche, Mannheim, Germany) was incorporated into the DNA probes and detection with anti‐DIG‐FITC (11‐207‐741‐910, Roche, Mannheim, Germany) or streptavidin‐Cy3 (S6402, Sigma, Deisenhofen, Germany), respectively. Chromosome numbers of *P. ginseng* are based on previous studies (Waminal *et al*., 2012; Waminal *et al*. 2016). Images were captured with an Olympus BX53 fluorescence microscope equipped with a Leica DFC365 FS CCD camera and processed using Cytovision ver. 7.2 (Leica Microsystems, Wetzlar, Germany). We performed further image enhancements using Adobe Photoshop CC.

### Accession numbers

The genome assembly, annotations and other multi‐omics data used in this study are available at our ginseng genome database ( http://ginsengdb.snu.ac.kr/) (Jayakodi *et al*., 2018), and all sequence data were deposited to the National Agricultural Biotechnology Information Center (NG‐0858‐000001~NG‐0858‐009845) ( http://nabic.rda.go.kr) (Seol *et al*., 2016). Chloroplast and 45S nrDNA genomes used in this study can be accessed in GenBank for *P. ginseng* (KM088019, KM036295), *P. quinquefolius* (KM088018, KM036297), *P. notoginseng* (KP036468, KT380921), *P. japonicus* (KP036469, KT380920), *P. vietnamensis* (KP036470, KT380922), *P. stipuleanatus* (KX247147, MF091695), *P. trifolius* (MF100782, MF099781), *A. elata* (KT153023, KT380919), *E. sessiliflorus* (KT153019, KT380924), *D. morbifera* (KR136270, KT380923) and *D. carota* (NC_008325, MF185182).

## Conflict of interest

The authors declare no conflict of interest.

## Author contributions

TJY conceived and supervised the research. NHK and SCL designed the experiments and managed particular components of the project. BSC and YY performed genome assembly, scaffolding and repeat annotation. WJ and NHK conducted assembly validation. MJ annotated the protein‐coding and noncoding genes and performed functional annotation for protein‐coding genes and other bioinformatics analysis. SCL, JGI and YCK contributed to RNA sample preparation and gene family analysis. JL and NEW performed repetitive analysis. NVB, KK and JYP performed chloroplast and rDNA assembly and phylogenomic analysis. ML, LK and DY constructed the genome‐scale metabolic network and assigned genes to each pathway. NEW and HK performed FISH analysis. YSL, HSP, HJK, and NVB induced the adventitious roots and treated MeJA and constructed library for RNA‐Seq. SP, HL, JK, ISK, ES, YIP, MSS, YL and HUK participated in gene family analysis. KBK and SHS profiled the metabolite data. MJ, NHK, SCL, NEW, NVB, AHP, DYL and TJY wrote the manuscript. DC, SK, DSP, DYH, CK, THL, HSK, YMK, DCY, RAW and AHP organized the manuscript.

## Supporting information


**Figure S1** Integrated pipeline for genome annotation (IPGA).
**Figure S2** Number of coding exons (CDS) comparison between plant species.
**Figure S3** Alternative splicing (AS) events in *P. ginseng*.
**Figure S4** The Ks distribution of paralog gene pairs and orthologs of five dicot plants.
**Figure S5** An example of *zigzag* extension of scaffold sequence.
**Figure S6** Comparative analysis of four homoeologous blocks in *P. ginseng*.
**Figure S7** Chromosomal mapping of genic regions from two adjacent contiguous scaffolds.
**Figure S8** Chloroplast (cp) genome maps of *P. stipuleanatus* and *P. trifolius*.
**Figure S9** Dotplot and mimetic diagram between scaffolds of *P. ginseng* and *P. notoginseng*.
**Figure S10** Characterization of *PgCACTA*.
**Figure S11** Karyotype idiogram of *P. ginseng* showing repetitive elements previously described as well as the Pg167TR elements.
**Figure S12** Global metabolic map for *P. ginseng*.
**Figure S13** Heat map for major ginsenoside pathway genes and 11 differentially expressed UGTs in response to methyl jasmonate (MeJA) in *P. ginseng* cv. Cheongsun (CS) adventitious roots.
**Figure S14** Visualization of global metabolic changes based on RNA‐seq expression.
**Figure S15** The number of differentially expressed (DE) genes among drought, salt, cold and stress samples.
**Figure S16** A phylogenetic relationship of FAD genes.
**Figure S17** A phylogenetic relationship of CAB family genes.
**Figure S18** Expression profiling of CAB genes in *P. ginseng*.
**Figure S19** Classification and estimation of CAB orthologs gene copies.
**Figure S20** *P. ginseng* specific expansion of TF family genes.
**Figure S21** Chromosomal distribution of major *P. ginseng* REs in *P. ginseng* chromosomes.
**Figure S22** Estimation of LTR‐RT insertion time in *P. ginseng*.

**Table S1** Whole genome sequencing (WGS) data generated in this study.
**Table S2** Statistics of *P. ginseng* draft genome sequence ver.1.0.
**Table S3** Mapping status of mate‐pair reads.
**Table S4** Validation summary of the *P. ginseng* genome assembly using 13 BAC clones.
**Table S5** Statistics of BUSCO assessment of genome assembly and gene set prediction in *P. ginseng*.
**Table S6** Statistics of repetitive elements in *P. ginseng*.
**Table S7** Transcriptome data generated and used in this study.
**Table S8** Comparative gene metrics of *P. ginseng* gene models.
**Table S9** Functional annotations of protein coding genes.
**Table S10** GO enrichment analysis for genes containing AS.
**Table S11** Transcription factor (TF) genes identified in the *P. ginseng* genome and other 18 plant genomes, using iTAK 1.6b standalone sw ( http://bioinfo.bti.cornell.edu/cgi-bin/itak/index.cgi).
**Table S12** Transcriptional regulator (TR) genes identified in the *P. ginseng* genome and other 18 plant genomes, using iTAK 1.6b standalone sw ( http://bioinfo.bti.cornell.edu/cgi-bin/itak/index.cgi).
**Table S13** Protein kinase (PK) genes identified in the *P. ginseng* genome and other 18 plant genomes, using iTAK 1.6b standalone sw ( http://bioinfo.bti.cornell.edu/cgi-bin/itak/index.cgi).
**Table S14** Chloroplast genomes and 45S nrDNA sequences used for comparative analysis in this study.
**Table S15** Downstream genes involved in ginsenosides biosynthesis comparison with relative plant species.
**Table S16** Ginsenosides quantification between *P. ginseng* cultivars.
**Table S17** The number of members in FAD gene family in plant genomes.

**Data S1** List of metabolites and metabolic reactions in ginseng genome‐scale metabolic network.

## References

[pbi12926-bib-0001] Alamancos, G.P. , Pagès, A. , Trincado, J.L. , Bellora, N. and Eyras, E. (2015) Leveraging transcript quantification for fast computation of alternative splicing profiles. RNA, 21, 1521–1531. 26179515 10.1261/rna.051557.115 PMC4536314

[pbi12926-bib-0002] Baeg, I.H. and So, S.H. (2013) The world ginseng market and the ginseng (Korea). J. Ginseng Res. 37, 1–7. 23717152 10.5142/jgr.2013.37.1 PMC3659626

[pbi12926-bib-0003] Barbazuk, W.B. , Fu, Y. and McGinnis, K.M. (2008) Genome‐wide analyses of alternative splicing in plants: opportunities and challenges. Genome Res. 18, 1381–1392. 18669480 10.1101/gr.053678.106

[pbi12926-bib-0004] Benveniste, P. (2004) Biosynthesis and accumulation of sterols. Annu. Rev. Plant Biol. 55, 429–457. 15377227 10.1146/annurev.arplant.55.031903.141616

[pbi12926-bib-0005] Cao, S. , Zhou, X.R. , Wood, C.C. , Green, A.G. , Singh, S.P. , Liu, L. and Liu, Q. (2013) A large and functionally diverse family of Fad2 genes in safflower (*Carthamus tinctorius* L.). BMC Plant Biol. 13, 5. 23289946 10.1186/1471-2229-13-5 PMC3554562

[pbi12926-bib-0006] Caspi, R. , Altman, T. , Billington, R. , Dreher, K. , Foerster, H. , Fulcher, C.A. , Holland, T.A. *et al* (2014) The MetaCyc database of metabolic pathways and enzymes and the BioCyc collection of Pathway/Genome Databases. Nucleic Acids Res. 42, D459–D471. 24225315 10.1093/nar/gkt1103 PMC3964957

[pbi12926-bib-0007] Chang, A. , Schomburg, I. , Placzek, S. , Jeske, L. , Ulbrich, M. , Xiao, M. , Sensen, C.W. *et al* (2014) BRENDA in 2015: exciting developments in its 25th year of existence. Nucleic Acids Res. 43, D439–D496. 25378310 10.1093/nar/gku1068 PMC4383907

[pbi12926-bib-0008] Cho, I.H. (2012) Effects of *Panax ginseng* in neurodegenerative diseases. J. Ginseng Res. 36, 342. 23717136 10.5142/jgr.2012.36.4.342 PMC3659610

[pbi12926-bib-0009] Choi, H.I. , Kim, N.H. , Lee, J. , Choi, B.S. , Do Kim, K. , Park, J.Y. , Lee, S.C. *et al* (2013) Evolutionary relationship of *Panax ginseng* and *P. quinquefolius* inferred from sequencing and comparative analysis of expressed sequence tags. Genet. Resour. Crop Evol. 60, 1377–1387.

[pbi12926-bib-0010] Choi, H.I. , Waminal, N.E. , Park, H.M. , Kim, N.H. , Choi, B.S. , Park, M. , Choi, D. *et al* (2014) Major repeat components covering one‐third of the ginseng (*Panax ginseng* C.A. Meyer) genome and evidence for allotetraploidy. Plant J. 77, 906–916. 24456463 10.1111/tpj.12441

[pbi12926-bib-0011] Chou, K.C. and Shen, H.B. (2010) Plant‐mPLoc: a top‐down strategy to augment the power for predicting plant protein subcellular localization. PLoS One, 5, e11335. 20596258 10.1371/journal.pone.0011335 PMC2893129

[pbi12926-bib-0012] Christensen, L.P. (2009) Ginsenosides: chemistry, biosynthesis, analysis, and potential health effects. Adv. Food Nutr. Res. 55, 1–99. 18772102 10.1016/S1043-4526(08)00401-4

[pbi12926-bib-0076] Court, W.E. (2000) Ginseng: The Genus Panax. Amsterdam, Netherlands: Hardwood Academic Publishers.

[pbi12926-bib-0013] Cock, J.M. , Sterck, L. , Rouzé, P. , Scornet, D. , Allen, A.E. , Amoutzias, G. , Anthouard, V. *et al* (2010) The Ectocarpus genome and the independent evolution of multicellularity in brown algae. Nature, 465, 617–621. 20520714 10.1038/nature09016

[pbi12926-bib-0014] Dai, X. and Zhao, P.X. (2011) psRNATarget: a plant small RNA target analysis server. Nucleic Acids Res. 39, W155–W159. 21622958 10.1093/nar/gkr319 PMC3125753

[pbi12926-bib-0015] Dillies, M.A. , Rau, A. , Aubert, J. , Hennequet‐Antier, C. , Jeanmougin, M. , Servant, N. , Keime, C. *et al* (2013) A comprehensive evaluation of normalization methods for Illumina high‐throughput RNA sequencing data analysis. Brief. Bioinform. 14, 671–683. 22988256 10.1093/bib/bbs046

[pbi12926-bib-0016] Drummond, A.J. and Rambaut, A. (2007) BEAST: Bayesian evolutionary analysis by sampling trees. BMC Evol. Biol. 7, 214. 17996036 10.1186/1471-2148-7-214 PMC2247476

[pbi12926-bib-0017] Fedoroff, N.V. and Bennetzen, J.L. (2013). Transposons, genomic shock, and genome evolution Plant Transposons and Genome Dynamics in Evolution (Fedoroff N.V., ed.), pp. 181–201. Ames, IA: Wiley‐Blackwell.

[pbi12926-bib-0018] Gao, Y.D. , Harris, A. , Zhou, S.D. and He, X.J. (2013) Evolutionary events in Lilium (including Nomocharis, Liliaceae) are temporally correlated with orogenies of the Q–T plateau and the Hengduan Mountains. Mol. Phylogenet. Evol. 68, 443–460. 23665039 10.1016/j.ympev.2013.04.026

[pbi12926-bib-0019] Gao, D. , Zhao, D. , Abernathy, B. , Iwata‐Otsubo, A. , Herrera‐Estrella, A. , Jiang, N. and Jackson, S.A. (2016) Dynamics of a novel highly repetitive CACTA family in common bean (*Phaseolus vulgaris*). G3 (Bethesda), 6, 2091–2101. 27185400 10.1534/g3.116.028761 PMC4938662

[pbi12926-bib-0020] Grabherr, M.G. , Haas, B.J. , Yassour, M. , Levin, J.Z. , Thompson, D.A. , Amit, I. , Adiconis, X. *et al* (2011) Full‐length transcriptome assembly from RNA‐Seq data without a reference genome. Nat. Biotechnol. 29, 644–652. 21572440 10.1038/nbt.1883 PMC3571712

[pbi12926-bib-0021] Haas, B.J. , Salzberg, S.L. , Zhu, W. , Pertea, M. , Allen, J.E. , Orvis, J. , White, O. *et al* (2008) Automated eukaryotic gene structure annotation using EVidenceModeler and the Program to Assemble Spliced Alignments. Genome Biol. 9, R7. 18190707 10.1186/gb-2008-9-1-r7 PMC2395244

[pbi12926-bib-0022] Han, J.Y. , In, J.G. , Kwon, Y.S. and Choi, Y.E. (2010) Regulation of ginsenoside and phytosterol biosynthesis by RNA interferences of squalene epoxidase gene in *Panax ginseng* . Phytochemistry, 71, 36–46. 19857882 10.1016/j.phytochem.2009.09.031

[pbi12926-bib-0023] Han, J.Y. , Kim, H.J. , Kwon, Y.S. and Choi, Y.E. (2011) The Cyt P450 enzyme CYP716A47 catalyzes the formation of protopanaxadiol from dammarenediol‐II during ginsenoside biosynthesis in *Panax ginseng* . Plant Cell Physiol. 52, 2062–2073. 22039120 10.1093/pcp/pcr150

[pbi12926-bib-0024] Han, J.Y. , Hwang, H.S. , Choi, S.W. , Kim, H.J. and Choi, Y.E. (2012) Cytochrome P450 CYP716A53v2 catalyzes the formation of protopanaxatriol from protopanaxadiol during ginsenoside biosynthesis in *Panax ginseng* . Plant Cell Physiol. 53, 1535–1545. 22875608 10.1093/pcp/pcs106

[pbi12926-bib-0025] Hong, C. , Lee, S. , Park, J. , Plaha, P. , Park, Y. , Lee, Y. , Choi, J. *et al* (2004) Construction of a BAC library of Korean ginseng and initial analysis of BAC‐end sequences. Mol. Genet. Genomics, 271, 709–716. 15197578 10.1007/s00438-004-1021-9

[pbi12926-bib-0026] Iorizzo, M. , Ellison, S. , Senalik, D. , Zeng, P. , Satapoomin, P. , Huang, J. , Bowman, M. *et al* (2016) A high‐quality carrot genome assembly provides new insights into carotenoid accumulation and asterid genome evolution. Nat. Genet. 48, 657–666. 27158781 10.1038/ng.3565

[pbi12926-bib-0027] Jang, W. , Kim, N.H. , Lee, J. , Waminal, N.E. , Lee, S.C. , Jayakodi, M. , Choi, H.I. *et al* (2017) A glimpse of *Panax ginseng* genome structure revealed from Ten BAC clone sequences obtained by SMRT sequencing platform. Plant Breed. Biotechnol. 5, 25–35.

[pbi12926-bib-0100] Jayakodi, M. , Choi, B.S. , Lee, S.C. , Kim, N.H. , Park, J.Y. , Jang, W. , Lakshmanan, M. , et al. (2018) Ginseng genome database: an open‐access platform for genomics of Panax ginseng. BMC Plant Biol. 18, 62 10.1186/s12870-018-1282-9 29649979 PMC5898050

[pbi12926-bib-0028] Jia, L. and Zhao, Y. (2009) Current evaluation of the millennium phytomedicine–ginseng (I): etymology, pharmacognosy, phytochemistry, market and regulations. Curr. Med. Chem. 19, 2475–2484. 10.2174/092986709788682146 PMC2752963 19601793

[pbi12926-bib-0029] Jiang, M. , Liu, J. , Quan, X. , Quan, L. and Wu, S. (2016) Different chilling stresses stimulated the accumulation of different types of ginsenosides in *Panax ginseng* cells. Acta Physiol. Plant. 38, 210.

[pbi12926-bib-0030] Jung, H.J. , Choi, H. , Lim, H.W. , Shin, D. , Kim, H. , Kwon, B. , Lee, J.E. *et al* (2012) Enhancement of anti‐inflammatory and antinociceptive actions of red ginseng extract by fermentation. J. Pharm. Pharmacol. 64, 756–762. 22471373 10.1111/j.2042-7158.2012.01460.x

[pbi12926-bib-0031] Kalendar, R. , Tanskanen, J. , Immonen, S. , Nevo, E. and Schulman, A.H. (2000) Genome evolution of wild barley (*Hordeum spontaneum*) by BARE‐1 retrotransposon dynamics in response to sharp microclimatic divergence. Proc. Natl Acad. Sci. USA, 97, 6603–6607. 10823912 10.1073/pnas.110587497 PMC18673

[pbi12926-bib-0032] Kanehisa, M. , Goto, S. , Sato, Y. , Furumichi, M. and Tanabe, M. (2012) KEGG for integration and interpretation of large‐scale molecular data sets. Nucleic Acids Res. 40, D109–D114. 22080510 10.1093/nar/gkr988 PMC3245020

[pbi12926-bib-0033] Khodakovskaya, M. , McAvoy, R. , Peters, J. , Wu, H. and Li, Y. (2006) Enhanced cold tolerance in transgenic tobacco expressing a chloroplast ω‐3 fatty acid desaturase gene under the control of a cold‐inducible promoter. Planta, 223, 1090–1100. 16292565 10.1007/s00425-005-0161-4

[pbi12926-bib-0034] Kim, N.H. , Choi, H.I. , Ahn, I.O. and Yang, T.J. (2012) EST‐SSR marker sets for practical authentication of all nine registered ginseng cultivars in Korea. J. Ginseng Res. 36, 298–307. 23717131 10.5142/jgr.2012.36.3.298 PMC3659598

[pbi12926-bib-0035] Kim, Y.J. , Jeon, J.N. , Jang, M.G. , Oh, J.Y. , Kwon, W.S. , Jung, S.K. and Yang, D.C. (2014) Ginsenoside profiles and related gene expression during foliation in *Panax ginseng* Meyer. J. Ginseng Res. 38, 66–72. 24558313 10.1016/j.jgr.2013.11.001 PMC3915334

[pbi12926-bib-0036] Kim, D. , Langmead, B. and Salzberg, S.L. (2015a) HISAT: a fast spliced aligner with low memory requirements. Nat. Methods, 12, 357–360. 25751142 10.1038/nmeth.3317 PMC4655817

[pbi12926-bib-0037] Kim, K. , Lee, S.C. , Lee, J. , Lee, H.O. , Joh, H.J. , Kim, N.H. , Park, H.S. *et al* (2015b) Comprehensive survey of genetic diversity in chloroplast genomes and 45S nrDNAs within *Panax ginseng* species. PLoS One, 10, e0117159. 26061692 10.1371/journal.pone.0117159 PMC4465672

[pbi12926-bib-0038] Kim, K. , Lee, S.C. , Lee, J. , Yu, Y. , Yang, K. , Choi, B.S. , Koh, H.J. *et al* (2015c) Complete chloroplast and ribosomal sequences for 30 accessions elucidate evolution of Oryza AA genome species. Sci. Rep. 5, 15655. 26506948 10.1038/srep15655 PMC4623524

[pbi12926-bib-0039] Kim, Y.J. , Zhang, D. and Yang, D.C. (2015d) Biosynthesis and biotechnological production of ginsenosides. Biotechnol. Adv. 33, 717–735. 25747290 10.1016/j.biotechadv.2015.03.001

[pbi12926-bib-0040] Kim, K. , Lee, S.C. and Yang, T.J. (2016a) The complete chloroplast genome sequence of *Dendropanax morbifera* (Leveille). Mitochondrial DNA A, 27, 2923–2924. 10.3109/19401736.2015.1060442 26153746

[pbi12926-bib-0041] Kim, K. , Lee, S.C. , Lee, J. , Kim, N.H. , Jang, W. and Yang, T.J. (2016b) The complete chloroplast genome sequence of *Panax quinquefolius* (L.). Mitochondrial DNA A DNA Mapp. Seq. Anal. 27, 3033–3034. 26162051 10.3109/19401736.2015.1063121

[pbi12926-bib-0042] Kim, K. , Nguyen, V.B. , Dong, J.Z. , Wang, Y. , Park, J.Y. , Lee, S.C. and Yang, T.J. (2017) Evolution of the Araliaceae family inferred from complete chloroplast genomes and 45S nrDNAs of 10 Panax‐related species. Sci. Rep. 7, 4917. In press. 28687778 10.1038/s41598-017-05218-y PMC5501832

[pbi12926-bib-0043] Kumar, V.S. , Dasika, M.S. and Maranas, C.D. (2007) Optimization based automated curation of metabolic reconstructions. BMC Bioinformatics, 8, 1. 17584497 10.1186/1471-2105-8-212 PMC1933441

[pbi12926-bib-0044] Lakshmanan, M. , Lim, S.H. , Mohanty, B. , Kim, J.K. , Ha, S.H. and Lee, D.L. (2015) Unraveling the light‐specific metabolic and regulatory signatures of rice through combined *in silico* modeling and multi‐omics analysis. Plant Physiol. 169, 3002–3020. 26453433 10.1104/pp.15.01379 PMC4677915

[pbi12926-bib-0045] Lee, J. , Waminal, N.E. , Choi, H.I. , Perumal, S. , Lee, S.C. , Nguyen, V.B. , Jang, W. *et al* (2017a) Rapid amplification of four retrotransposon families promoted speciation and genome size expansion in the genus Panax. Sci. Rep. 7, 9045. 28831052 10.1038/s41598-017-08194-5 PMC5567358

[pbi12926-bib-0046] Lee, Y. , Park, H.S. , Lee, D.K. , Jayakodi, M. , Kim, N.H. , Koo, H.J. , Lee, S.C. *et al* (2017b) Integrated transcriptomic and metabolomic analysis of five *Panax ginseng* cultivars reveals the dynamics of ginsenoside biosynthesis. Front. Plant Sci. 8, 1048. 28674547 10.3389/fpls.2017.01048 PMC5474932

[pbi12926-bib-0047] Li, H. and Durbin, R. (2009) Fast and accurate short read alignment with Burrows–Wheeler transform. Bioinformatics, 25, 1754–1760. 19451168 10.1093/bioinformatics/btp324 PMC2705234

[pbi12926-bib-0048] Li, R. and Wen, J. (2016) Phylogeny and diversification of Chinese Araliaceae based on nuclear and plastid DNA sequence data. J. Syst. Evol. 54, 453–467.

[pbi12926-bib-0049] Li, L. , Stoeckert, C.J. and Roos, D.S. (2003) OrthoMCL: identification of ortholog groups for eukaryotic genomes. Genome Res. 13, 2178–2189. 12952885 10.1101/gr.1224503 PMC403725

[pbi12926-bib-0101] Li, W. and Godzik, A. (2006) Cd‐hit: a fast program for clustering and comparing large sets of protein or nucleotide sequences. Bioinformatics 22, 1658‐1659. 16731699 10.1093/bioinformatics/btl158

[pbi12926-bib-0050] Luo, R. , Liu, B. , Xie, Y. , Li, Z. , Huang, W. , Yuan, J. , He, G. *et al* (2012) SOAPdenovo2: an empirically improved memory‐efficient short‐read *de novo* assembler. Gigascience, 1, 18. 23587118 10.1186/2047-217X-1-18 PMC3626529

[pbi12926-bib-0051] Lyons, E. , Pedersen, B. , Kane, J. and Freeling, M. (2008) The value of nonmodel genomes and an example using SynMap within CoGe to dissect the hexaploidy that predates the rosids. Trop. Plant Biol. 1, 181–190.

[pbi12926-bib-0052] Marçais, G. and Kingsford, C. (2011) A fast, lock‐free approach for efficient parallel counting of occurrences of k‐mers. Bioinformatics, 27, 764–770. 21217122 10.1093/bioinformatics/btr011 PMC3051319

[pbi12926-bib-0053] Mathiyalagan, R. , Subramaniyam, S. , Natarajan, S. , Kim, Y.J. , Sun, M.S. , Kim, S.Y. , Kim, Y.J. *et al* (2013) Insilico profiling of microRNAs in Korean ginseng (*Panax ginseng* Meyer). J. Ginseng Res. 37, 227–247. 23717176 10.5142/jgr.2013.37.227 PMC3659641

[pbi12926-bib-0054] Oh, J.Y. , Kim, Y.J. , Jang, M.G. , Joo, S.C. , Kwon, W.S. , Kim, S.Y. , Jung, S.K. *et al* (2014) Investigation of ginsenosides in different tissues after elicitor treatment in *Panax ginseng* . J. Ginseng Res. 38, 270–277. 25379007 10.1016/j.jgr.2014.04.004 PMC4213849

[pbi12926-bib-0055] Pertea, M. , Pertea, G.M. , Antonescu, C.M. , Chang, T.C. , Mendell, J.T. and Salzberg, S.L. (2015) StringTie enables improved reconstruction of a transcriptome from RNA‐seq reads. Nat. Biotechnol. 33, 290–295. 25690850 10.1038/nbt.3122 PMC4643835

[pbi12926-bib-0056] Radad, K. , Gille, G. , Liu, L. and Rausch, W.D. (2006) Use of ginseng in medicine with emphasis on neurodegenerative disorders. J. Pharmacol. Sci. 100, 175–186. 16518078 10.1254/jphs.crj05010x

[pbi12926-bib-0057] Robinson, M.D. , McCarthy, D.J. and Smyth, G.K. (2010) edgeR: a Bioconductor package for differential expression analysis of digital gene expression data. Bioinformatics, 26, 139–140. 19910308 10.1093/bioinformatics/btp616 PMC2796818

[pbi12926-bib-0058] Román, Á. , Andreu, V. , Hernández, M.L. , Lagunas, B. , Picorel, R. , Martínez‐Rivas, J.M. and Alfonso, M. (2012) Contribution of the different omega‐3 fatty acid desaturase genes to the cold response in soybean. J. Exp. Bot. 63, 4973–4982. 22865909 10.1093/jxb/ers174 PMC3427996

[pbi12926-bib-0059] Schellenberger, J. , Que, R. , Fleming, R.M. , Thiele, I. , Orth, J.D. , Feist, A.M. , Zielinski, D.C. *et al* (2011) Quantitative prediction of cellular metabolism with constraint‐based models: the COBRA Toolbox v2. 0. Nat. Protoc. 6, 1290–1307. 21886097 10.1038/nprot.2011.308 PMC3319681

[pbi12926-bib-0060] Schwartz, S. , Zhang, Z. , Frazer, K.A. , Smit, A. , Riemer, C. , Bouck, J. , Gibbs, R. *et al* (2000) PipMaker—a web server for aligning two genomic DNA sequences. Genome Res. 10, 577–586. 10779500 10.1101/gr.10.4.577 PMC310868

[pbi12926-bib-0061] Schwartz, S. , Kent, W.J. , Smit, A. , Zhang, Z. , Baertsch, R. , Hardison, R.C. , Haussler, D. *et al* (2003) Human–mouse alignments with BLASTZ. Genome Res. 13, 103–107. 12529312 10.1101/gr.809403 PMC430961

[pbi12926-bib-0062] Seol, Y.J. , Lee, T.H. , Park, D.S. and Kim, C.K. (2016) NABIC: a new access portal to search, visualize, and share agricultural genomics data. Evol. Bioinf. Online, 12, 51. 10.4137/EBO.S34493 PMC4737523 26848255

[pbi12926-bib-0063] Shewfelt, R. (1992). Response of plant membranes to chilling and freezing. *Plant membranes* (Springer), pp. 192–219.

[pbi12926-bib-0064] Shi, W. , Wang, Y. , Li, J. , Zhang, H. and Ding, L. (2007) Investigation of ginsenosides in different parts and ages of *Panax ginseng* . Food Chem. 102, 664–668.

[pbi12926-bib-0065] Simão, F.A. , Waterhouse, R.M. , Ioannidis, P. , Kriventseva, E.V. and Zdobnov, E.M. (2015) BUSCO: assessing genome assembly and annotation completeness with single‐copy orthologs. Bioinformatics, 31, 3210–3212. 26059717 10.1093/bioinformatics/btv351

[pbi12926-bib-0066] Smit, A. and Hubley, R. (2010). RepeatModeler Open‐1.0. http://www.repeatmasker.org.

[pbi12926-bib-0067] Smit, A.F. , Hubley, R. and Green, P. (1996). RepeatMasker Open‐3.0.http://www.repeatmasker.org.

[pbi12926-bib-0068] Tamura, K. , Stecher, G. , Peterson, D. , Filipski, A. and Kumar, S. (2013) MEGA6: molecular evolutionary genetics analysis version 6.0. Mol. Biol. Evol. 30, 2725–2729. 24132122 10.1093/molbev/mst197 PMC3840312

[pbi12926-bib-0069] Tansakul, P. , Shibuya, M. , Kushiro, T. and Ebizuka, Y. (2006) Dammarenediol‐II synthase, the first dedicated enzyme for ginsenoside biosynthesis, in *Panax ginseng* . FEBS Lett. 580, 5143–5149. 16962103 10.1016/j.febslet.2006.08.044

[pbi12926-bib-0070] Thiele, I. and Palsson, B.Ø. (2010) A protocol for generating a high‐quality genome‐scale metabolic reconstruction. Nat. Protoc. 5, 93–121. 20057383 10.1038/nprot.2009.203 PMC3125167

[pbi12926-bib-0071] Thomashow, M.F. (1998) Role of cold‐responsive genes in plant freezing tolerance. Plant Physiol. 118, 1–8. 9733520 10.1104/pp.118.1.1 PMC1539187

[pbi12926-bib-0072] Thomashow, M.F. (1999) Plant cold acclimation: freezing tolerance genes and regulatory mechanisms. Annu. Rev. Plant Biol. 50, 571–599. 10.1146/annurev.arplant.50.1.571 15012220

[pbi12926-bib-0073] Waminal, N.E. , Choi, H.I. , Kim, N.H. , Jang, W. , Lee, J. , Park, J.Y. , Kim, H.H. *et al* (2016) A refined *Panax ginseng* karyotype based on an ultra‐high copy 167‐bp tandem repeat and ribosomal DNAs. J. Ginseng Res. 41, 469–476. 29021693 10.1016/j.jgr.2016.08.002 PMC5628329

[pbi12926-bib-0074] Waminal, N.E. , Park, H.M. , Ryu, K.B. , Kim, J.H. , Yang, T.J. and Kim, H.H. (2012) Karyotype analysis of *Panax ginseng* C.A.Meyer, 1843 (Araliaceae) based on rDNA loci and DAPI band distribution. Comp. Cytogenet. 6, 425–441. 24260682 10.3897/CompCytogen.v6i4.3740 PMC3834566

[pbi12926-bib-0075] Wang, Y. , Tang, H. , DeBarry, J.D. , Tan, X. , Li, J. , Wang, X. , Lee, T.H. *et al* (2012) MCScanX: a toolkit for detection and evolutionary analysis of gene synteny and collinearity. Nucleic Acids Res. 40, e49. 22217600 10.1093/nar/gkr1293 PMC3326336

[pbi12926-bib-0077] Wong, A.S. , Che, C.M. and Leung, K.W. (2015) Recent advances in ginseng as cancer therapeutics: a functional and mechanistic overview. Nat. Prod. Rep. 32, 256–272. 25347695 10.1039/c4np00080c

[pbi12926-bib-0078] Xiao, D. , Yue, H. , Xiu, Y. , Sun, X. , Wang, Y. and Liu, S. (2015) Accumulation characteristics and correlation analysis of five ginsenosides with different cultivation ages from different regions. J. Ginseng Res. 39, 338–344. 26869826 10.1016/j.jgr.2015.03.004 PMC4593784

[pbi12926-bib-0079] Xie, J.T. , Mehendale, S.R. , Li, X. , Quigg, R. , Wang, X. , Wang, C.Z. , Wu, J.A. *et al* (2005) Anti‐diabetic effect of ginsenoside Re in ob/ob mice. Biochem. Biophys. Acta. 1740, 319–325. 15949698 10.1016/j.bbadis.2004.10.010

[pbi12926-bib-0080] Yang, Z. (2007) PAML 4: phylogenetic analysis by maximum likelihood. Mol. Biol. Evol. 24, 1586–1591. 17483113 10.1093/molbev/msm088

[pbi12926-bib-0102] Zdobnov, E.M. and Apweiler, R. (2001) InterProScan‐an integration platform for the signature‐recognition methods in InterPro. Bioinformatics 17, 847‐848. 11590104 10.1093/bioinformatics/17.9.847

[pbi12926-bib-0081] Zhang, D. , Li, W. , Xia, E.H. , Zhang, Q.J. , Liu, Y. , Zhang, Y. , Tong, Y. *et al* (2017) The medicinal herb *Panax notoginseng* genome provides insights into ginsenoside biosynthesis and genome evolution. Mol. Plant, 10, 903–907. 28315473 10.1016/j.molp.2017.02.011

[pbi12926-bib-0082] Zheng, S.D. , Wu, H.J. and Wu, D.l. (2012) Roles and mechanisms of ginseng in protecting heart. Chin. J. Integr. Med. 18, 548–555. 22772919 10.1007/s11655-012-1148-1

[pbi12926-bib-0083] Zheng, Y. , Jiao, C. , Sun, H. , Rosli, H.G. , Pombo, M.A. , Zhang, P. , Banf, M. *et al* (2016) iTAK: a program for genome‐wide prediction and classification of plant transcription factors, transcriptional regulators, and protein kinases. Mol. Plant, 9, 1667–1670. 27717919 10.1016/j.molp.2016.09.014

