# Supplementary material for: Genome and evolution of the shade‐requiring medicinal herb Panax ginseng
Source: Plant Biotechnol J. 2018 May 25;16(11):1904–17. doi: 10.1111/pbi.12926 (PMC6181221; doi:10.1111/pbi.12926)
Supplement: Supplementary file 1 — Figure S1 Integrated pipeline for genome annotation (IPGA). Figure S2 Number of coding exons (CDS) comparison between plant species. Figure S3 Alternative splicing (AS) events in P. ginseng. Figure S4 The Ks distribution of paralog gene pairs and orthologs of five dicot plants. Figure S5 An example of zigzag extension of scaffold sequence. Figure S6 Comparative analysis of four homoeologous blocks in P. ginseng. Figure S7 Chromosomal mapping of genic regions from two adjacent contiguous scaffolds. Figure S8 Chloroplast (cp) genome maps of P. stipuleanatus and P. trifolius. Figure S9 Dotplot and mimetic diagram between scaffolds of P. ginseng and P. notoginseng. Figure S10 Characterization of PgCACTA. Figure S11 Karyotype idiogram of P. ginseng showing repetitive elements previously described as well as the Pg167TR elements. Figure S12 Global metabolic map for P. ginseng. Figure S13 Heat map for major ginsenoside pathway genes and 11 differentially expressed UGTs in response to methyl jasmonate (MeJA) in P. ginseng cv. Cheongsun (CS) adventitious roots. Figure S14 Visualization of global metabolic changes based on RNA‐seq expression. Figure S15 The number of differentially expressed (DE) genes among drought, salt, cold and stress samples. Figure S16 A phylogenetic relationship of FAD genes. Figure S17 A phylogenetic relationship of CAB family genes. Figure S18 Expression profiling of CAB genes in P. ginseng. Figure S19 Classification and estimation of CAB orthologs gene copies. Figure S20 P. ginseng specific expansion of TF family genes. Figure S21 Chromosomal distribution of major P. ginseng REs in P. ginseng chromosomes. Figure S22 Estimation of LTR‐RT insertion time in P. ginseng. [file PBI-16-1904-s001.doc]

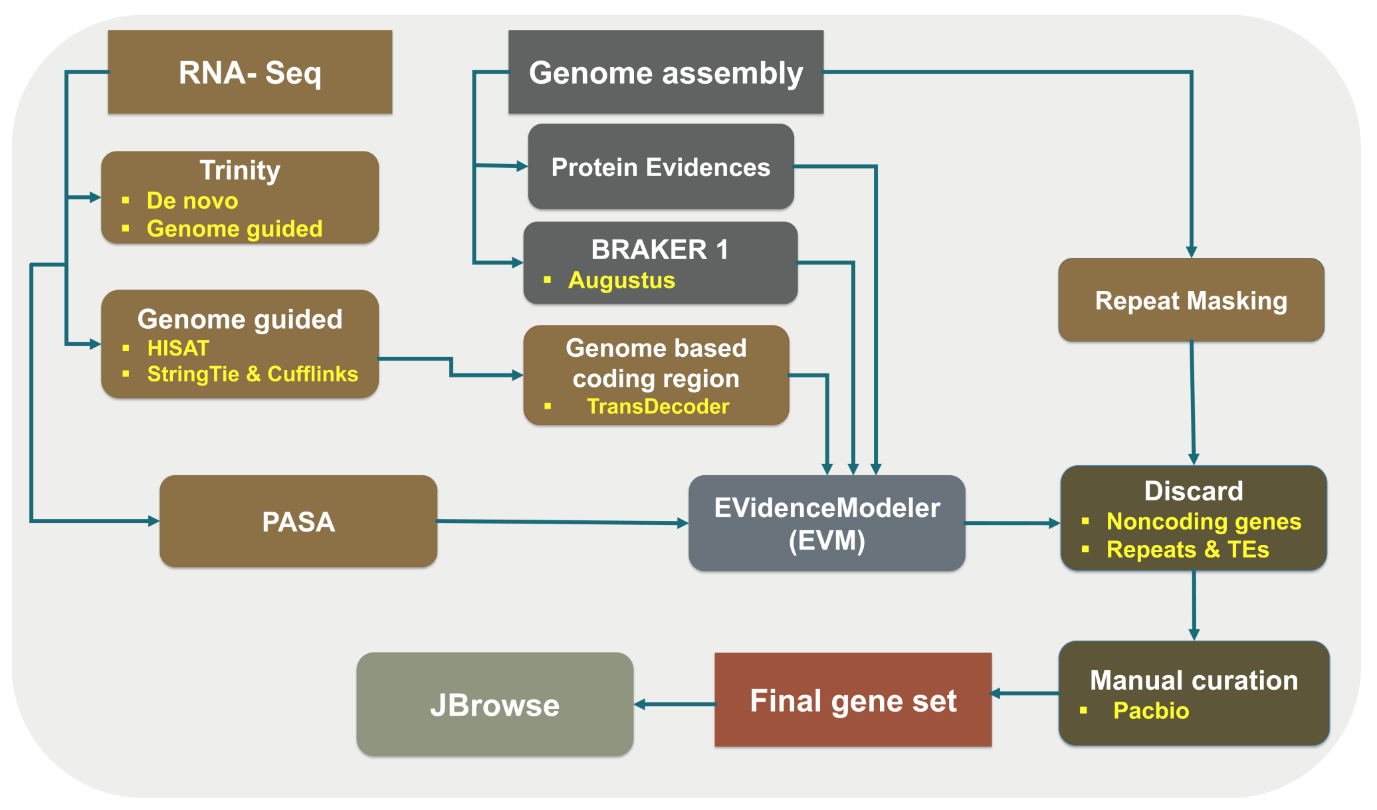


**Supplemental Figure 1**. **Integrated pipeline for genome annotation (IPGA).** The pipeline figure shows the methodology and programs used for *P. ginseng* genome annotation. To annotate the genic regions in *P. ginseng* draft genome, RNA-seq data generated from *P. ginseng* cv. ChP were assembled by both *de novo* and reference based methods and then resulting gene information was merged with *ab initio* and protein evidences to make consensus gene models. Further curation of gene model was done using PacBio transcriptome and then the curated gene set was used to construct *P. ginseng* genome browser.


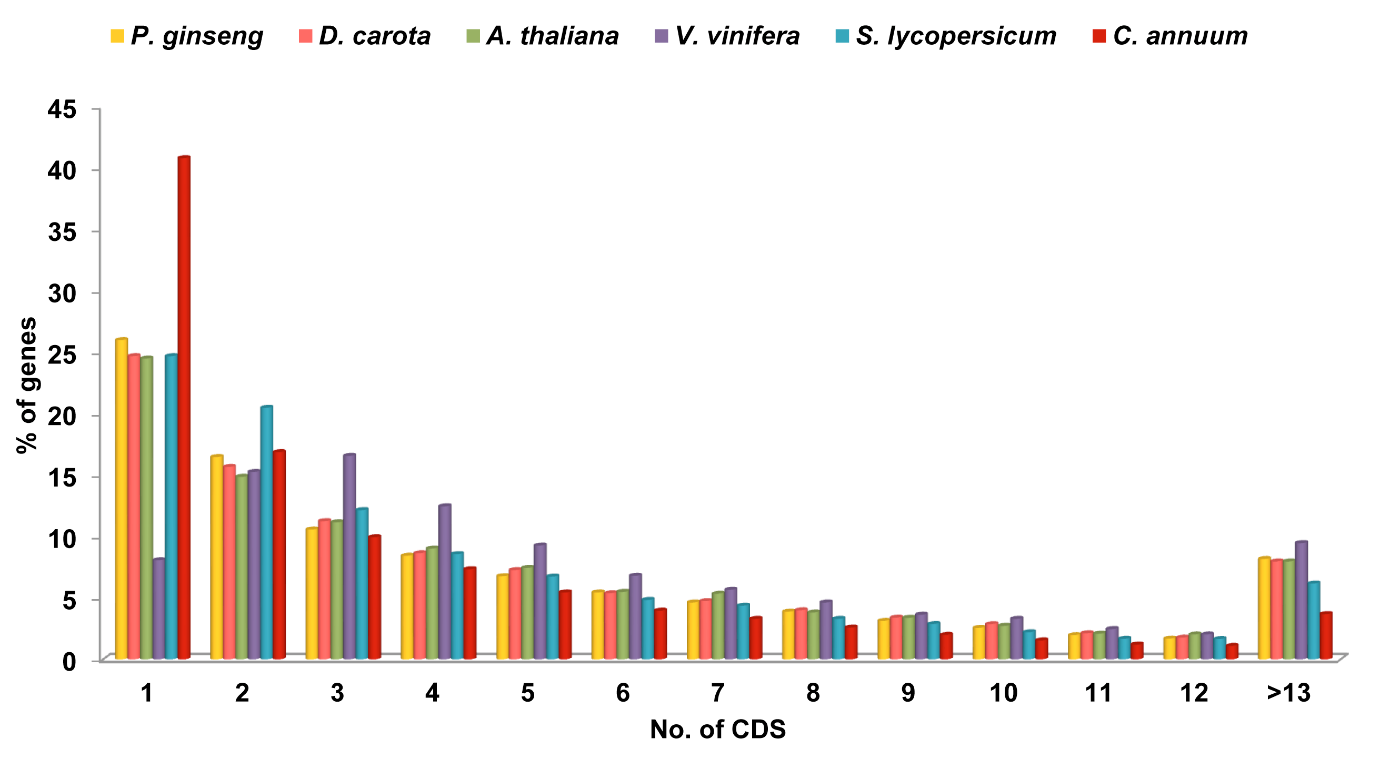


**Supplemental Figure 2. Number of coding exons (CDS) comparison between plant species.** A Python script was used to calculate number of CDS for each gene in the above species based on genome annotation file (gff).


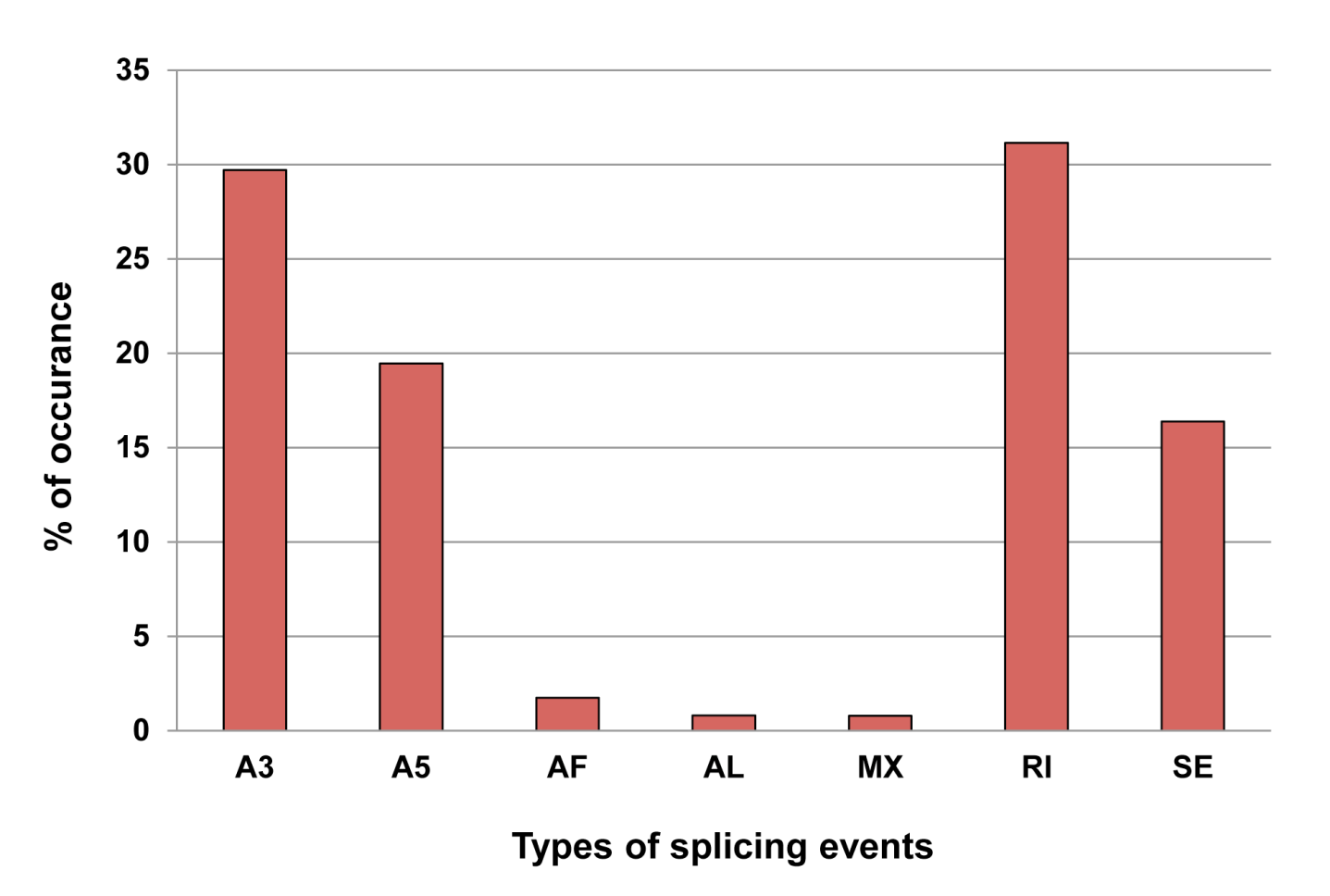


**Supplemental Figure 3**. **Alternative splicing (AS) events in *P. ginseng*.** The x-axis shows the types of splicing events including alternative 3’ splice-site (A3), alternative 5’ splice-site (A5), alternative first exon (AF), alternative last exon (AL), mutually exclusive exons (MX), retained intron (RI) and skipping exon (SE) and y-axis represents the percentage of occurrence of corresponding AS events in *P. ginseng*.


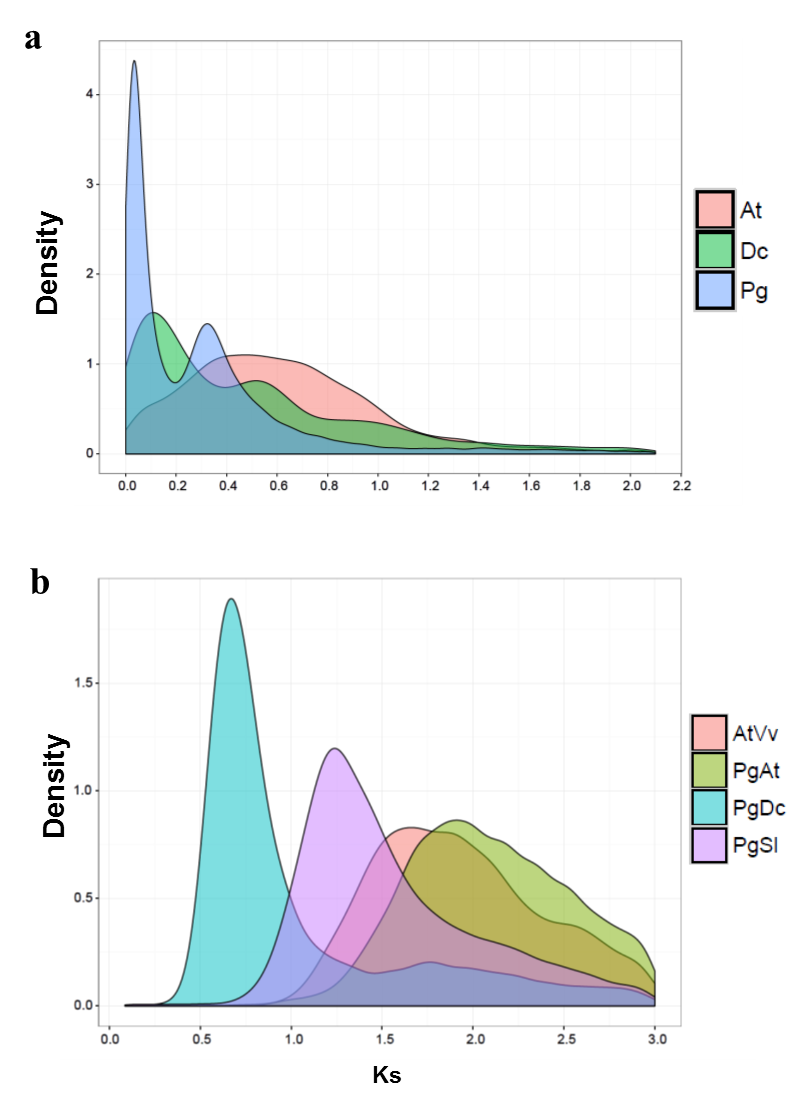


**Supplemental Figure 4. The Ks distribution of paralog gene pairs and orthologs of five dicot plants*.* a,** Density plot of Ks value using paralog gene pairs of Arabidopsis (At), carrot (Dc), and ginseng (Pg). **b**, Orthologous Ks density plot between ginseng and other plants including grapes (Vv), tomato (Sl).


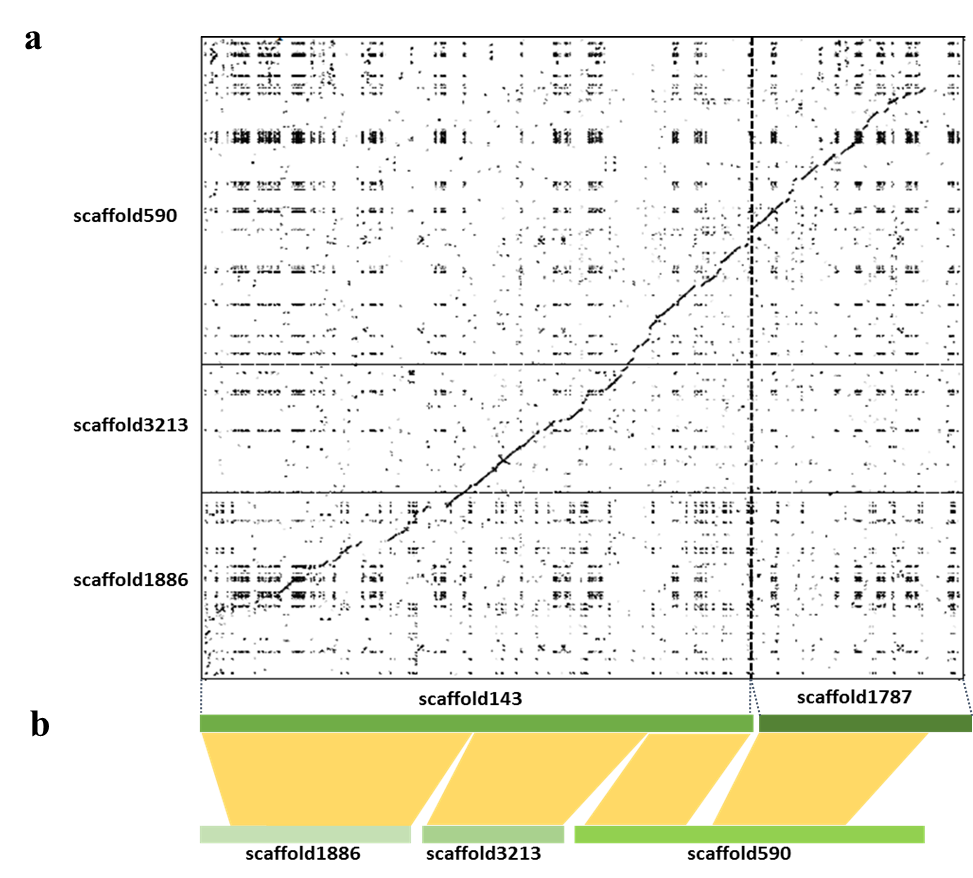


**Supplemental Figure 5. An example of *zig-zag* extension of scaffold sequence.** Scaffold sequences could be extended by counterpart scaffold sequences on the basis of synteny between paralogous scaffold sequences. **a**, A total of three scaffold sequences (Pg_scaffold00590, Pg_scaffold03213, and Pg_scaffold01886) showed sequence collinearity with two (Pg_scaffold00143 and Pg_scaffold01787) in v0.8 draft sequence. Pg_scaffold01886 and Pg_scaffold03213 were concatenated in v1.0 draft sequence. **b**, Mimetic diagram of two paralogous blocks by connection of the three and two sequences, respectively.


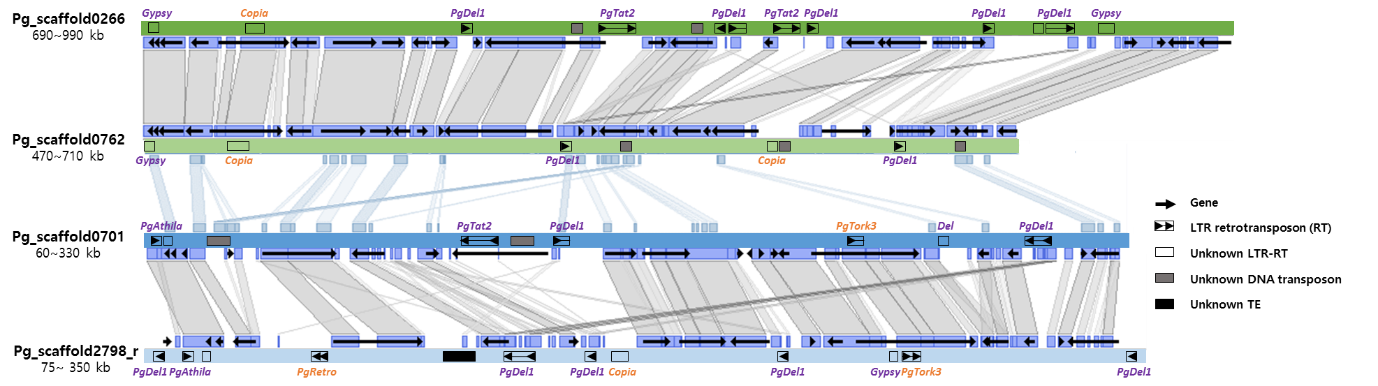


**Supplemental Figure 6. Comparative analysis of four homoeologous blocks in *P. ginseng*.** Homologous region between paralogs were identified with BLASTZ and repetitive elements were characterized with identified *P. ginseng* TEs and CENSOR program.


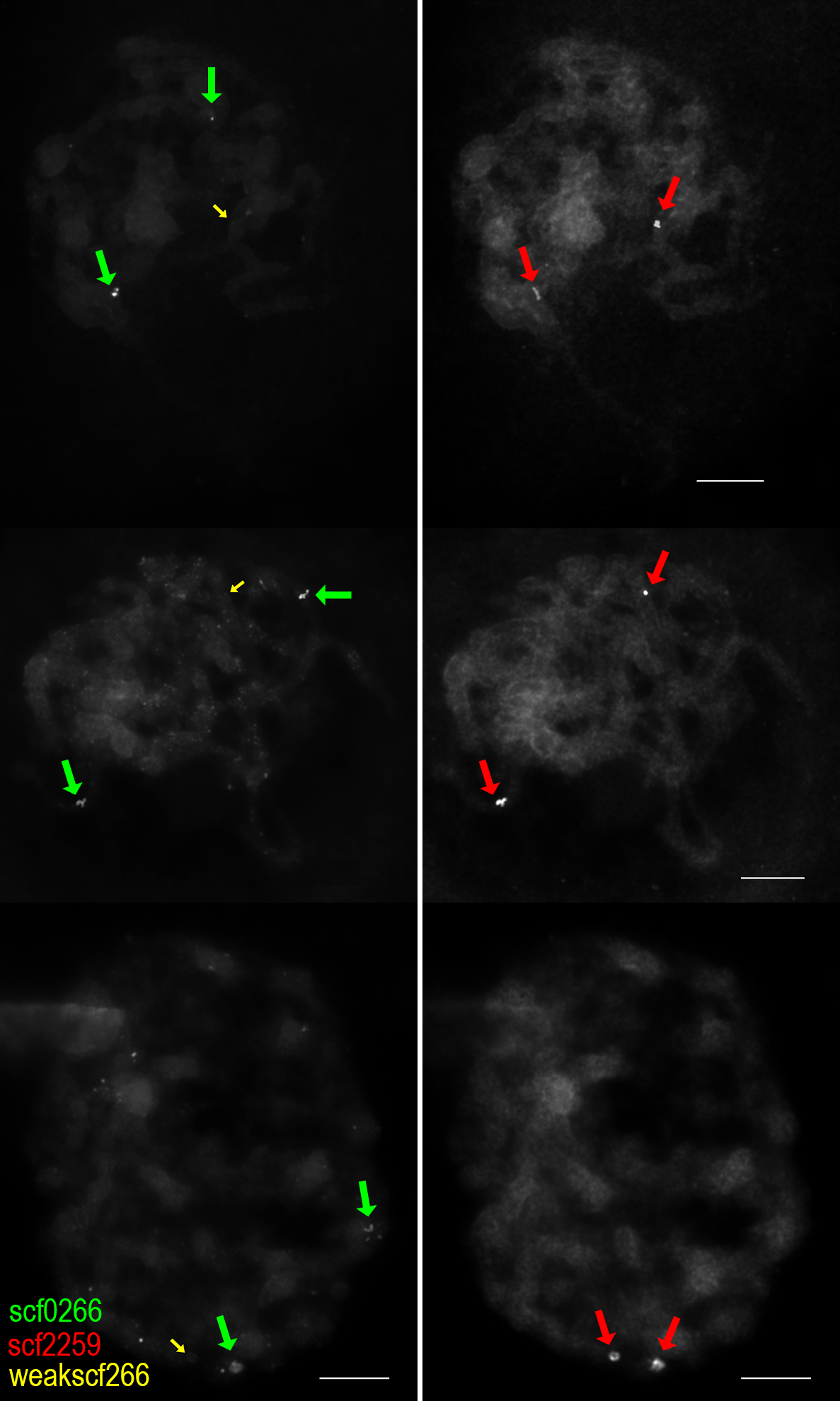


**Supplemental Figure 7. Chromosomal mapping of genic regions from two adjacent contiguous scaffolds.** Two intense clustered signals were observed for each pooled genic probes from Pg_scaffold0266 and Pg_scaffold2259, indicating duplicated genic blocks from each scaffold. In addition, a very weak Pg_scaffold0266 signal linked with Pg_scaffold2259 locus (yellow arrow), supports their linkage in one paralogous site.


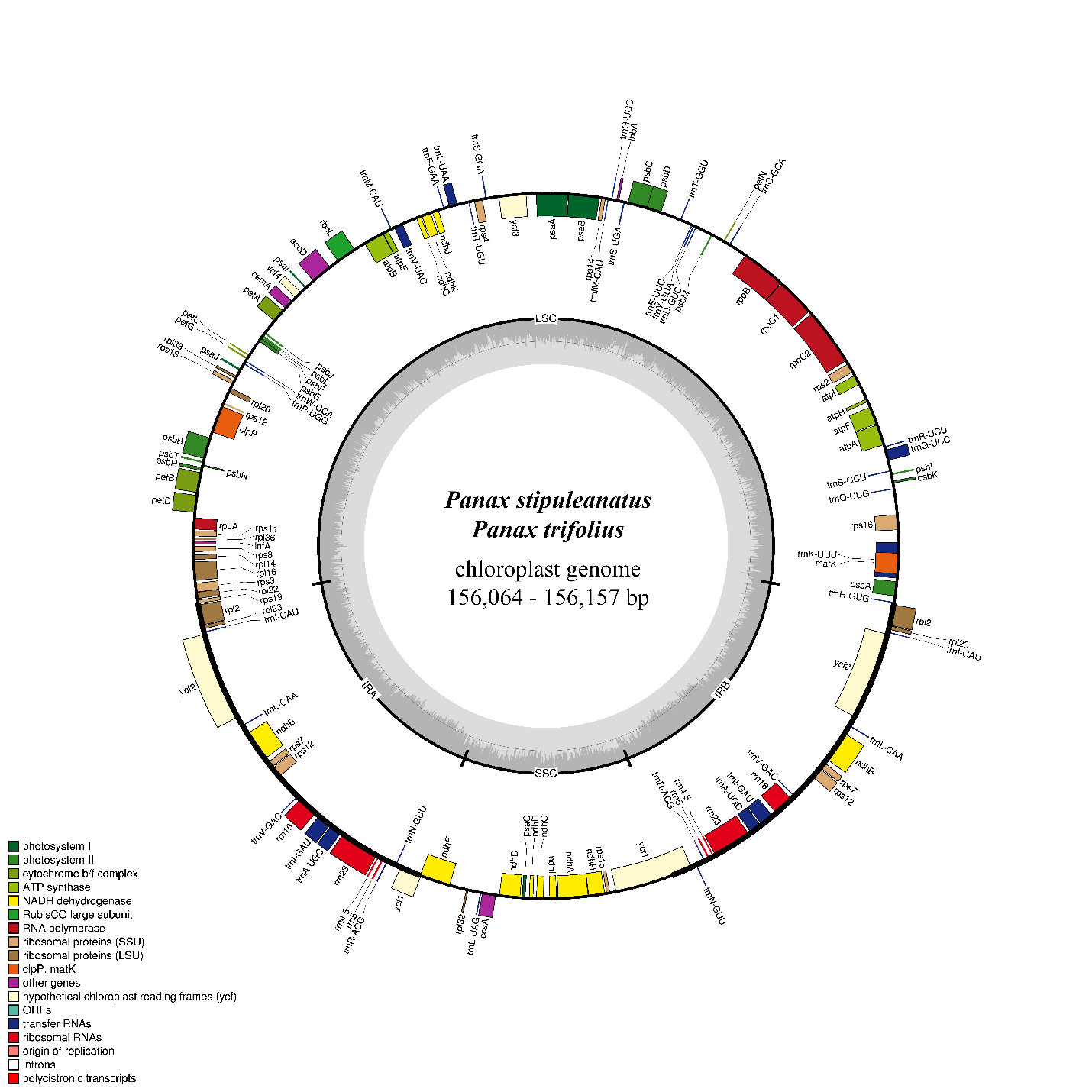


**Supplemental Figure 8. Chloroplast (cp) genome maps of *P. stipuleanatus* and *P. trifolius*.** Colored boxes are conserved chloroplast genes classified based on gene annotation. The complete cp genome sequence was generated by the dnaLCW method and annotated using the DOGMA program (http://dogma.ccbb.utexas.edu/). The map was prepared using OGDRAW (http://ogdraw.mpimp-golm.mpg.de/). Genes transcribed clockwise and counterclockwise are indicated on the outside and inside of the large circle, respectively.
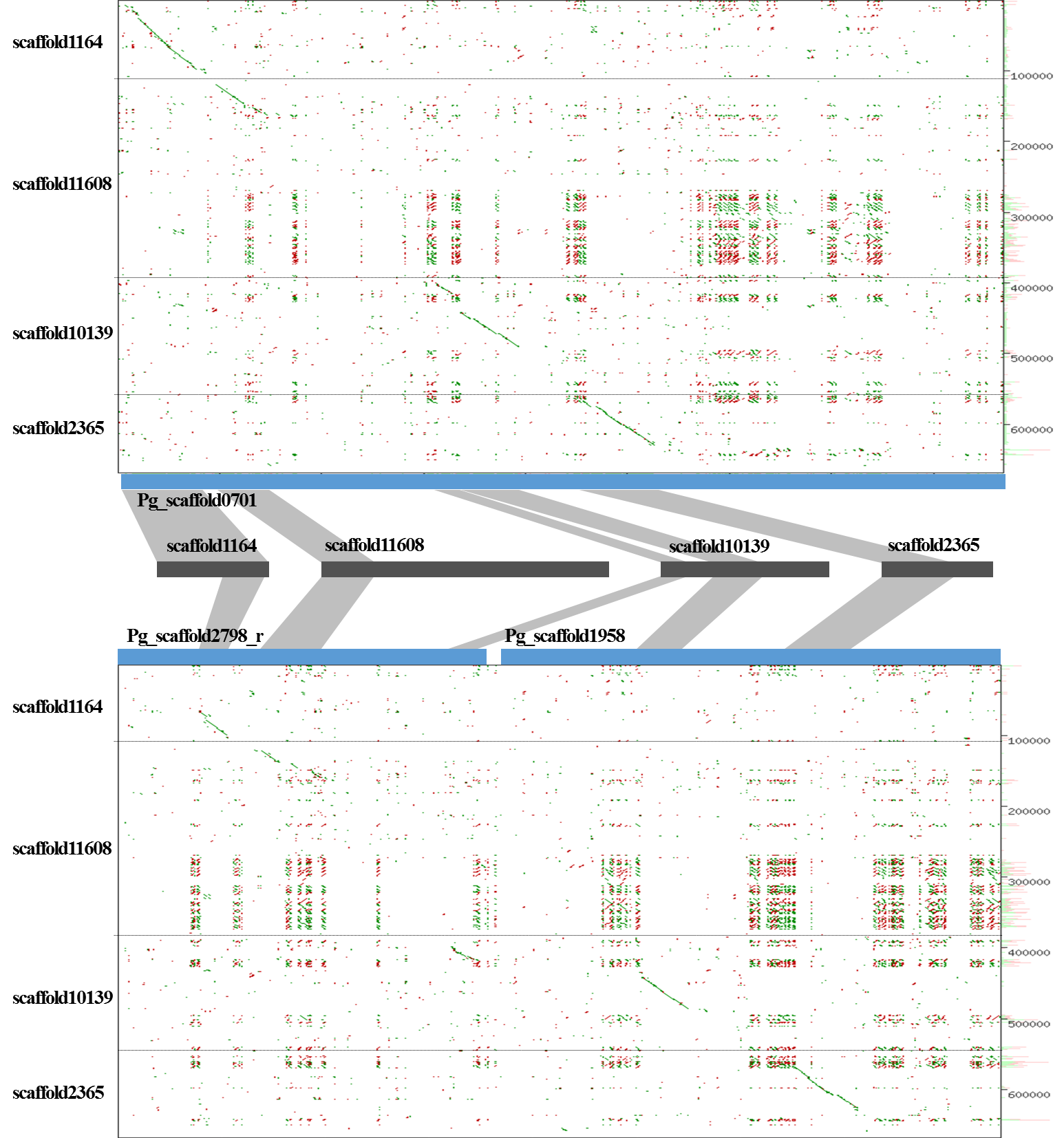


**Supplemental Figure 9**. **Dotplot and mimetic diagram between scaffolds of *P. ginseng* and *P. notoginseng.*** The homologous scaffolds of *P. notoginseng* was identified using BLASTN search against *P. ginseng* scaffolds. Dotplot was generated by YASS (<http://bioinfo.lifl.fr/yass/yass.php>) with default parameter.


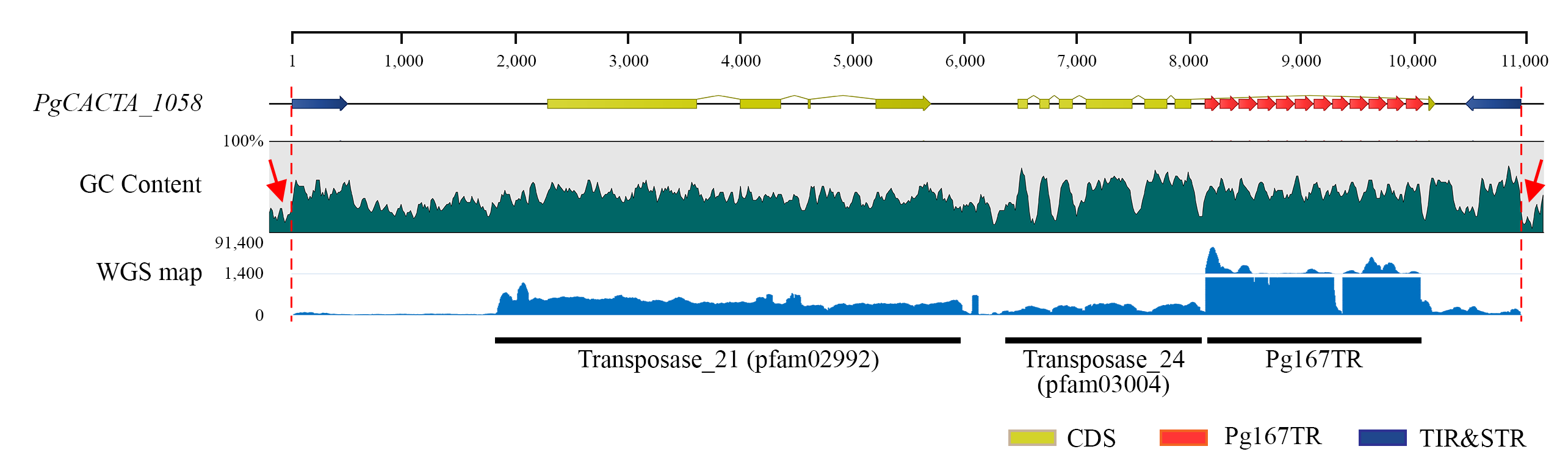


**Supplemental Figure 10. Characterization of *PgCACTA.*** Characterization of a full length putative autonomous PgCACTA_1058. Two CDS were predicted using *Z. mays* model in FGENESH gene prediction. The upstream CDS matched with a transposase_21 superfamily and a domain of unknown function (DUF), while the downstream CDS matched with a transposase_24 superfamily. WGS read mapping showed a relatively higher copy of transposase_21 than transposase_24 (WGS map). Pg167TR repeats were localized at the last intron of the transposase_24 CDS and revealed high abundance. Insertion sites (red arrows) of PgCACTA_1058 showed high AT composition relative to its internal region, indicative of relatively recent insertion and putative autonomy (GC Content).


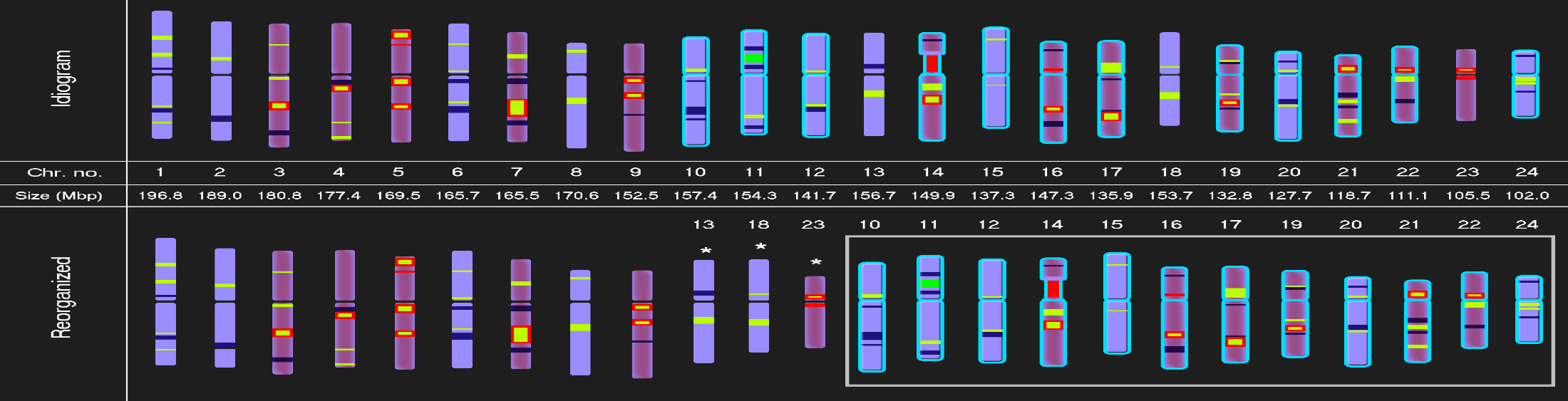


**Supplemental Figure 11. Karyotype idiogram of *P. ginseng* showing repetitive elements previously described as well as the Pg167TR elements.** Blue, green, red, and yellow bars indicate DAPI, 5S rDNA, 45S rDNA, and Pg167TR bands. Pg167TR bands with red borders indicate Pg167TRb. Purple and stroked chromosomes represent Pg167TR and *PgDel2*-rich (Choi et al, 2014) chromosomes. Pg167TRb loci localized in six out of 12 *PgDel2*-rich ginseng chromosomes. This brings the possibility that the ginseng genome was derived from ancient genome with six as basic chromosome number. Lower panel: After rearranging the chromosome based on the presence or absence of *PgDel2*, it is more apparent that chromosomes bearing the *PgDel2­* LTR retrotransposons are generally shorter than those without *PgDel2*. Bars= 10 μm .


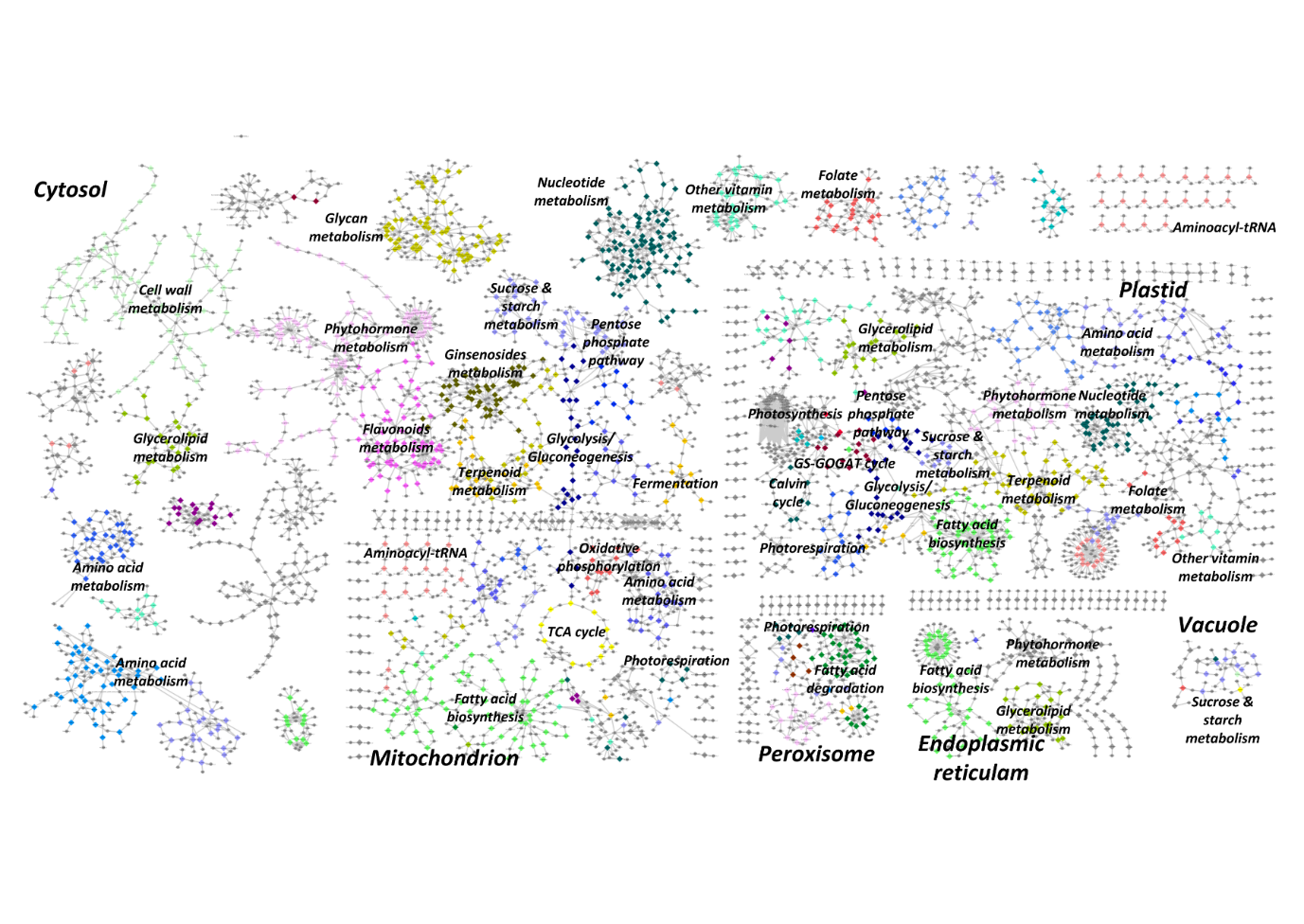


**Supplemental Figure 12. Global metabolic map for *P. ginseng*.** Metabolic reactions and their corresponding compartments generated from genome-scale metabolic network is represented with different colors.


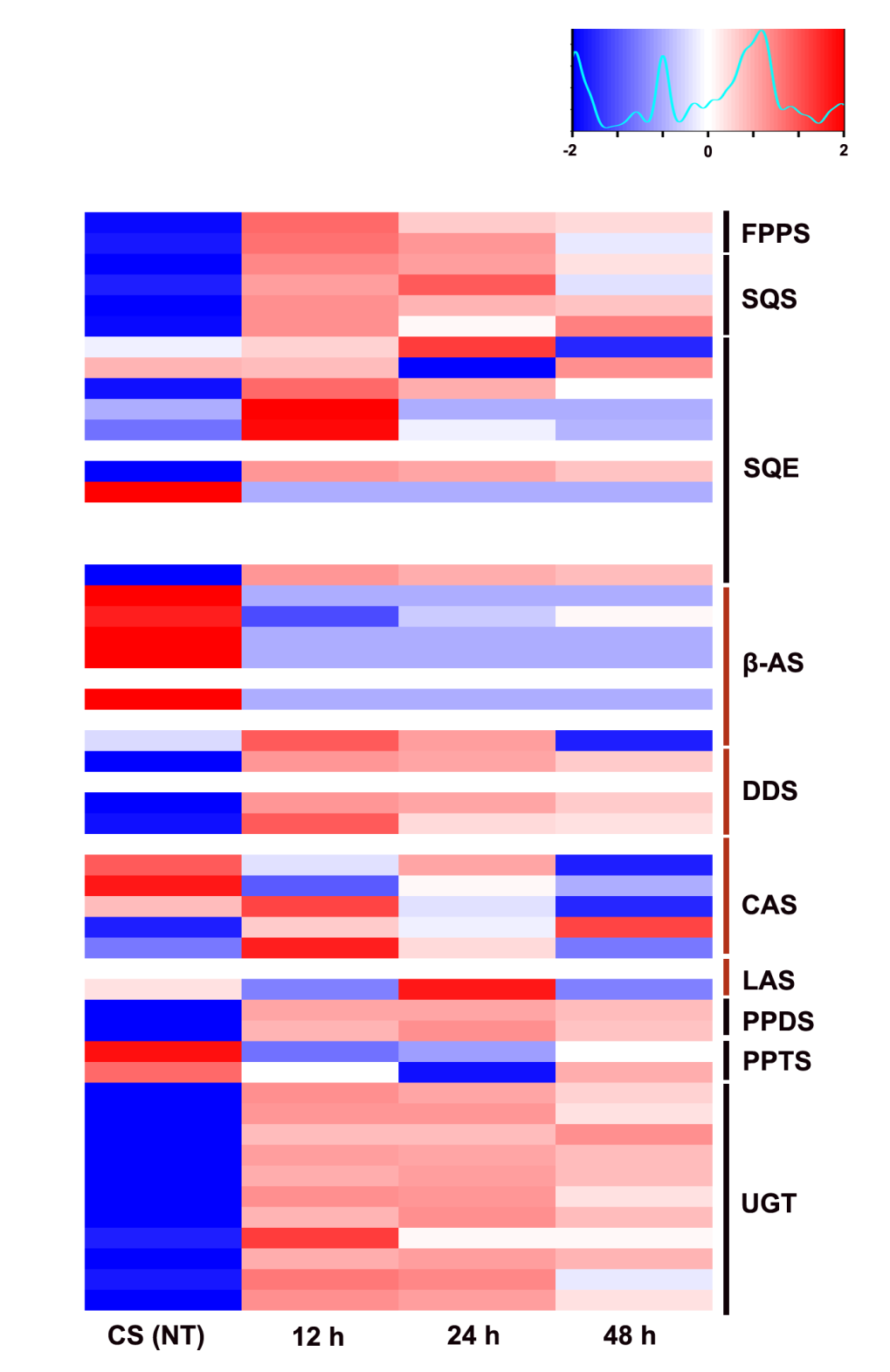


**Supplemental Figure 13. Heat map for major ginsenoside pathway genes and 11 differentially expressed UGTs in response to methyl jasmonate (MeJA) in *P. ginseng* cv. Cheongsun (CS) adventitious roots.** MeJA treatment was performed for 12 hours (h), 24 h and 48 h. Heatmap displays downstream genes and its corresponding expression pattern in comparison with control or not treated (NT). The OSC gene groups are represented in red color of vertical bar on the right side of the figure.


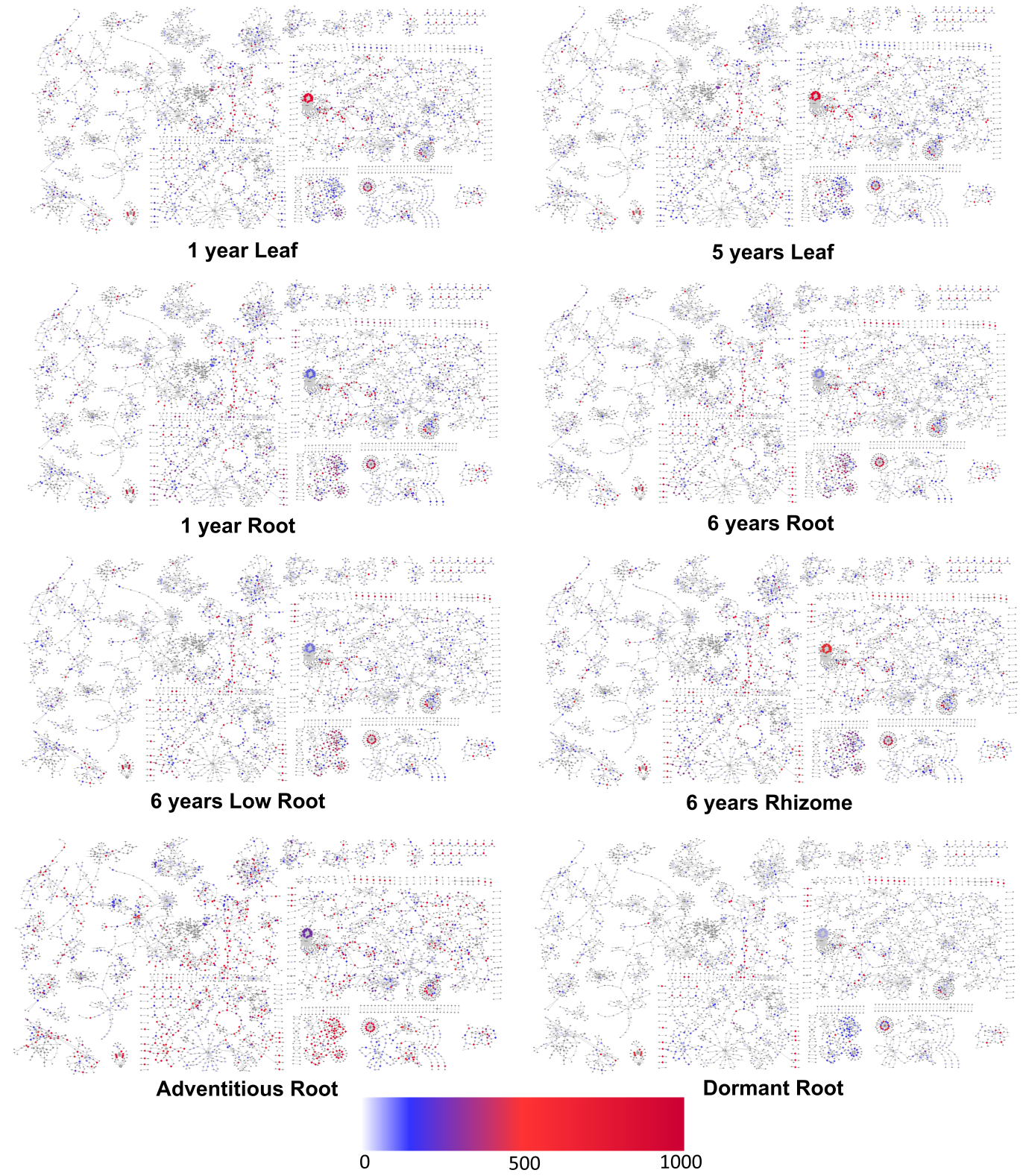


**Supplemental Figure 14. Visualization of global metabolic changes based on RNA-seq expression.** The RNA-seq expression data (FPKM) from 1-, 5-year old leaves, 1-, 6-year old main roots, lateral roots, rhizome and dormant roots, 4-weeks old adventitious roots were mapped to genome-scale metabolic networks and observed the metabolic changes depends on tissue and growth year.


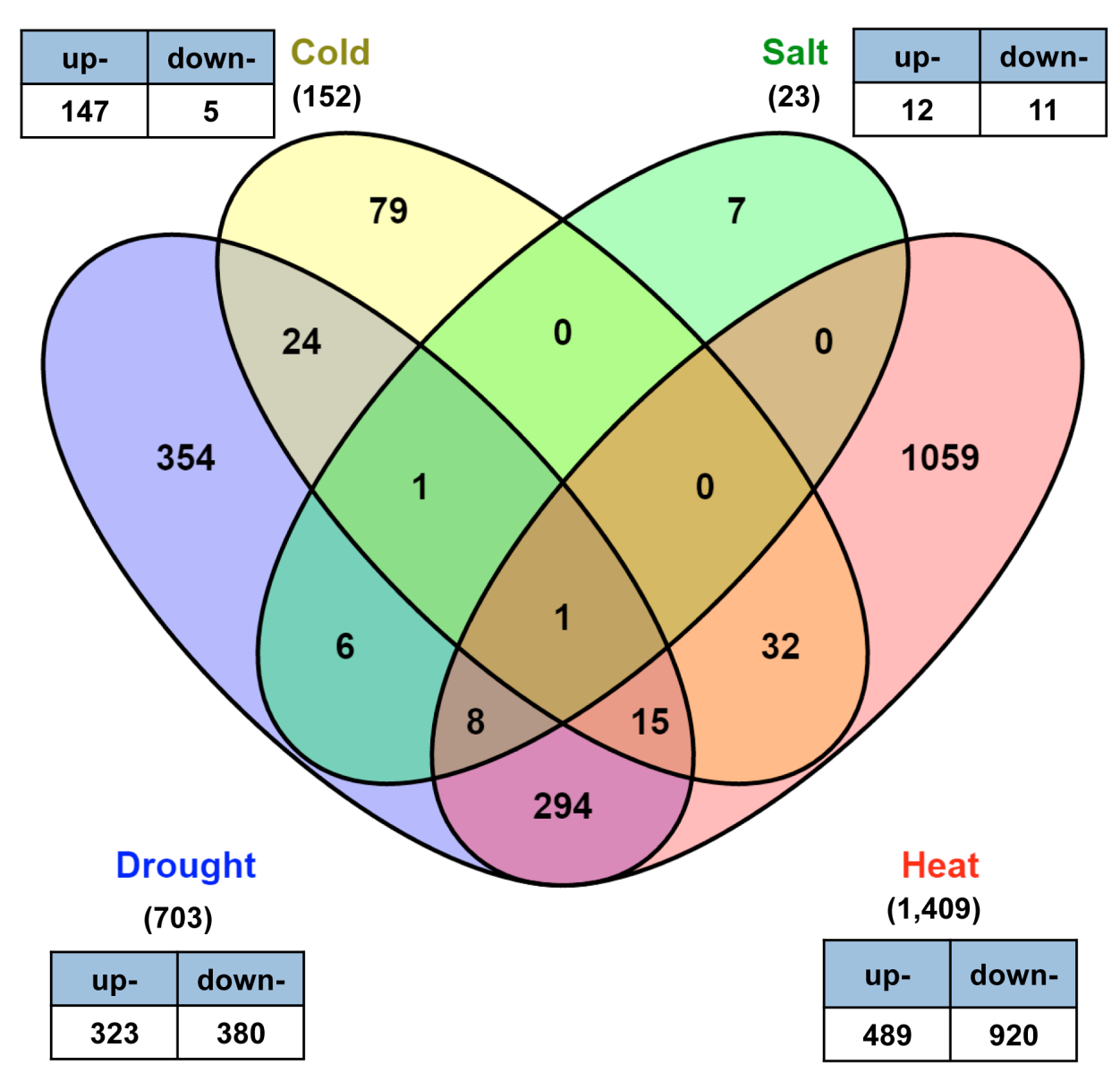


**Supplemental Figure 15**. **The number of differentially expressed (DE) genes among drought, salt, cold and stress samples.** A Venn diagram intersects the number of DE genes among abiotic stress along the number of up- and down-regulated genes. The abiotic samples include control plants (1-year old whole plants of leaves, stems and roots), salt (whole plants treated with 100 mM NaCl for 24 hr), cold (whole plants treated with cold (4°C) for 24 hr) and drought (whole plants treated with drought (air-drying) for 24 hr). For heat stress, leaves of plants (1-year old, cv. ChP) treated with heat (30°C) for 1 and 3 weeks.


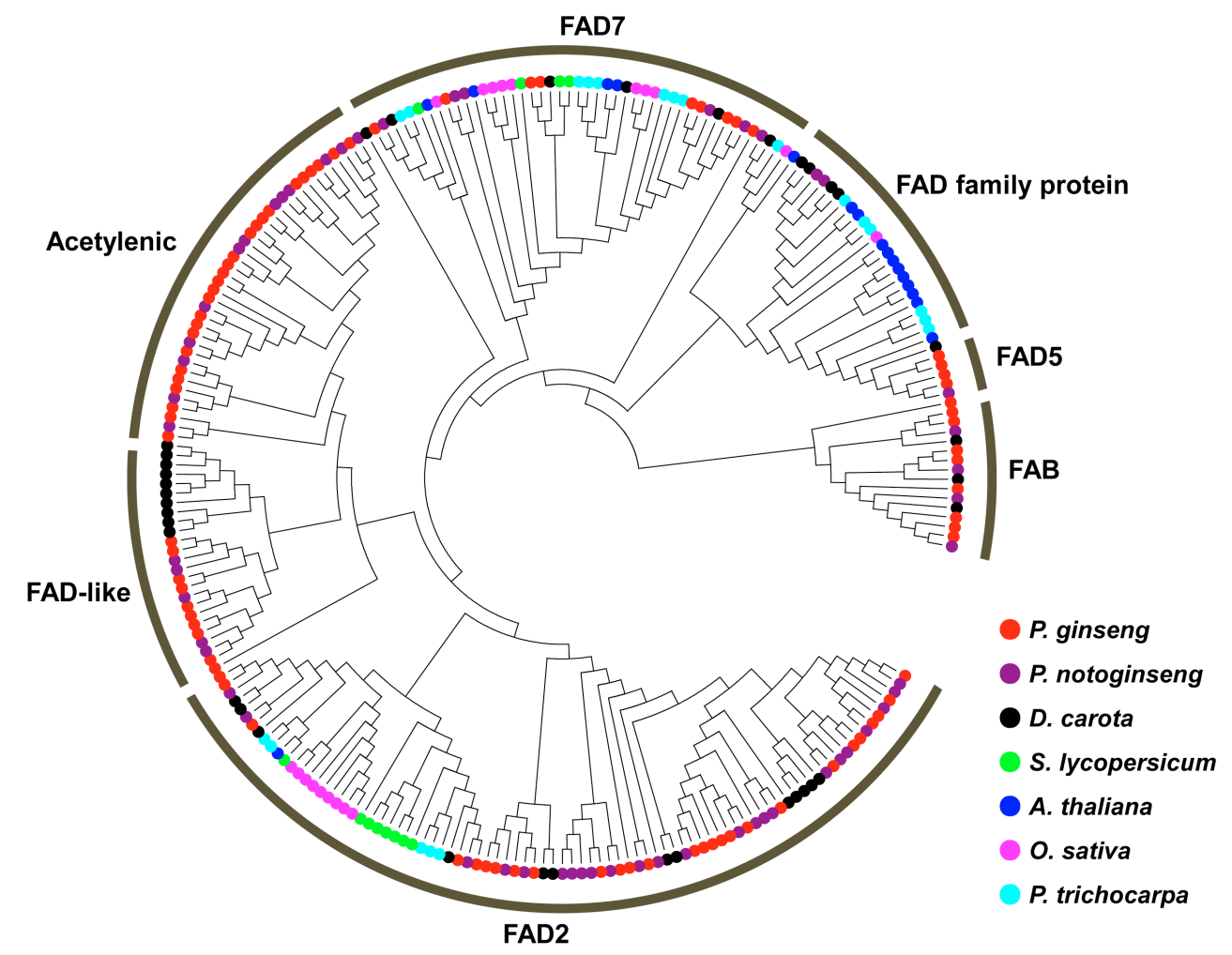


**Supplemental Figure 16. A phylogenetic relationship of FAD genes.** The red, purple, black, blue, green, pink and cyan color indicates the corresponding genes in the FAD family from Korean ginseng, Chinese ginseng, carrot, Arabidopsis, tomato, rice and poplar. The outer circle indicates the grouping of subgroup FADs including acetylenic, FAD-like, FAD2, FAD5, FAD7, FAD family protein and FAB.


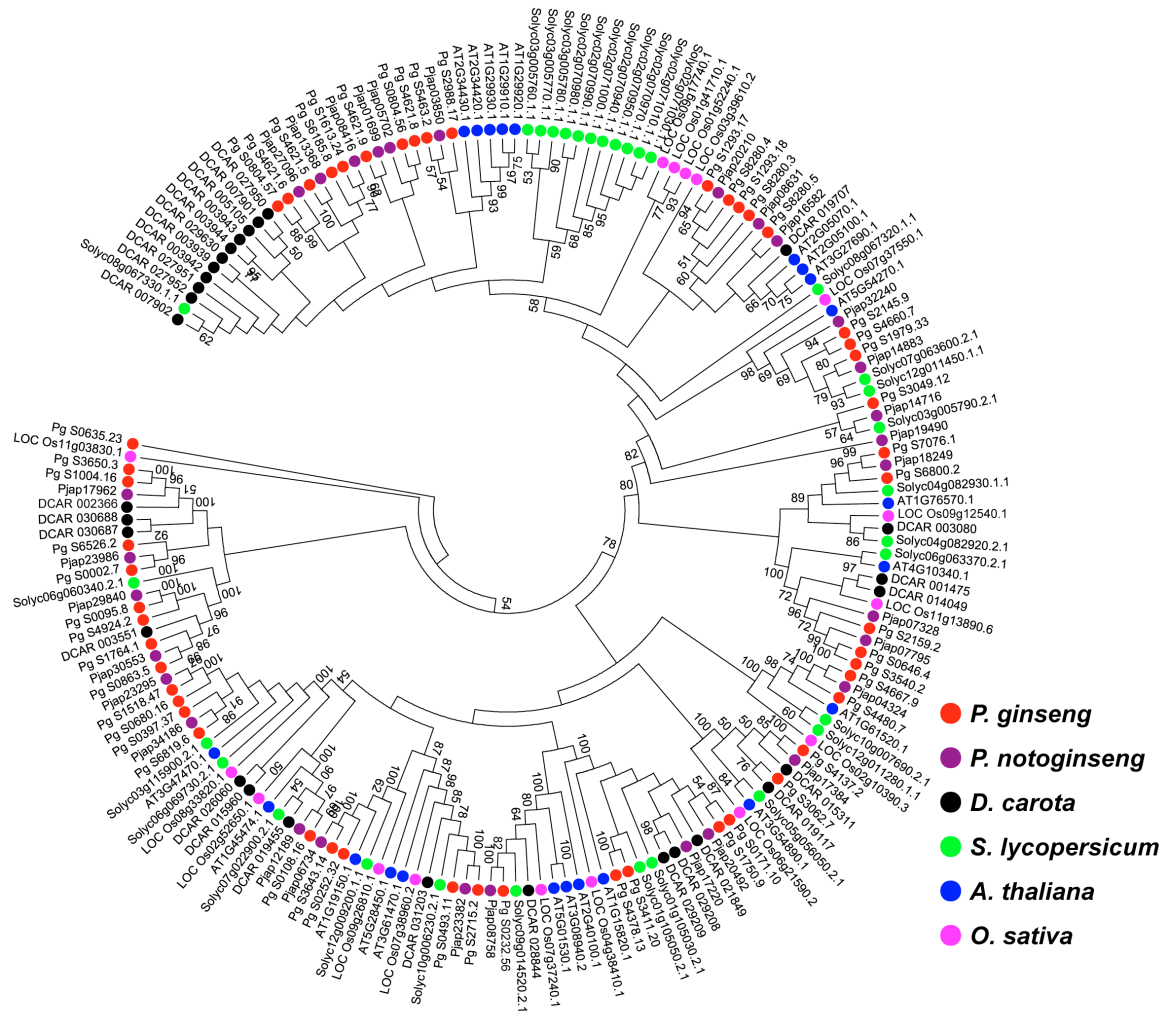


**Supplemental Figure 17**. **A phylogenetic relationship of CAB family genes.** The red, purple, black, blue, green and pink color indicates the corresponding genes in the CAB family from Korean ginseng, Chinese ginseng, carrot, arabidopsis, tomato and rice respectively.


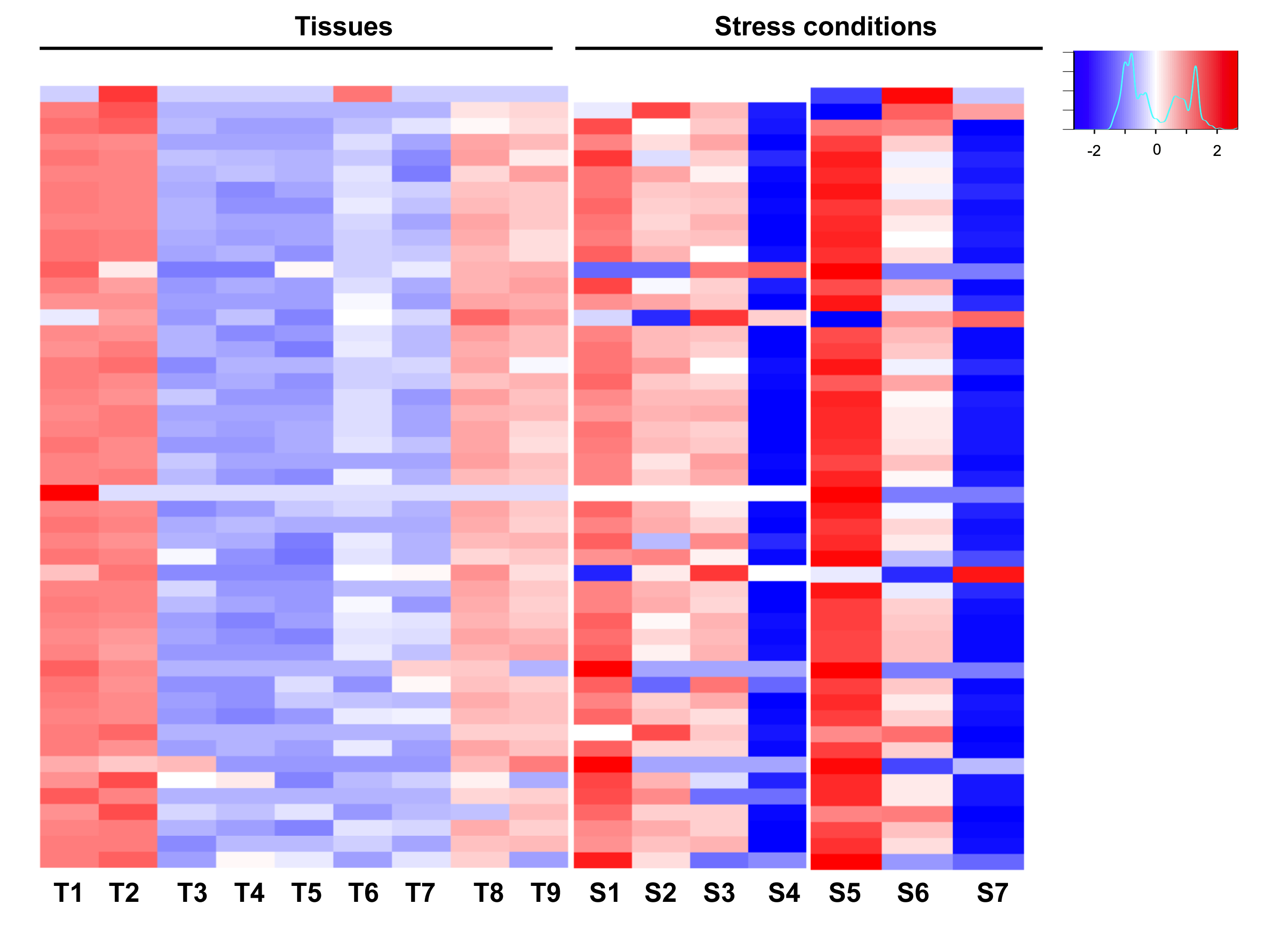


**Supplementary Figure** **18**. **Expression profiling of CAB genes** **in** ***P. ginseng***. Heatmap shows TMM normalized expression values of 49 CAB genes in ginseng**.** Expression in various tissues (T1: one-year old leaves, T2: five-year old leaves, T3: one-year old main body roots, T4: six-year old main body roots, T5: six-year old lateral roots, T6: six-year old rhizomes, T7: six-year old dormant roots, T8: flower, T9: six-year old stem) and abiotic stresses (S1: control plants (1-year old whole plants of leaves, stems and roots), S2: salt (whole plants treated with 100 mM NaCl for 24 hr), S3: cold (whole plants treated with cold (4°C) for 24 hr), S4: drought (whole plants treated with drought (air-drying) for 24 hr, S5: leaves of plants (1-year old, cv. ChP, Control), S6: leaves of plants treated with heat (30°C) for 1 week and S7: leaves of plants treated with heat (30°C) for 3 weeks) are depicted.


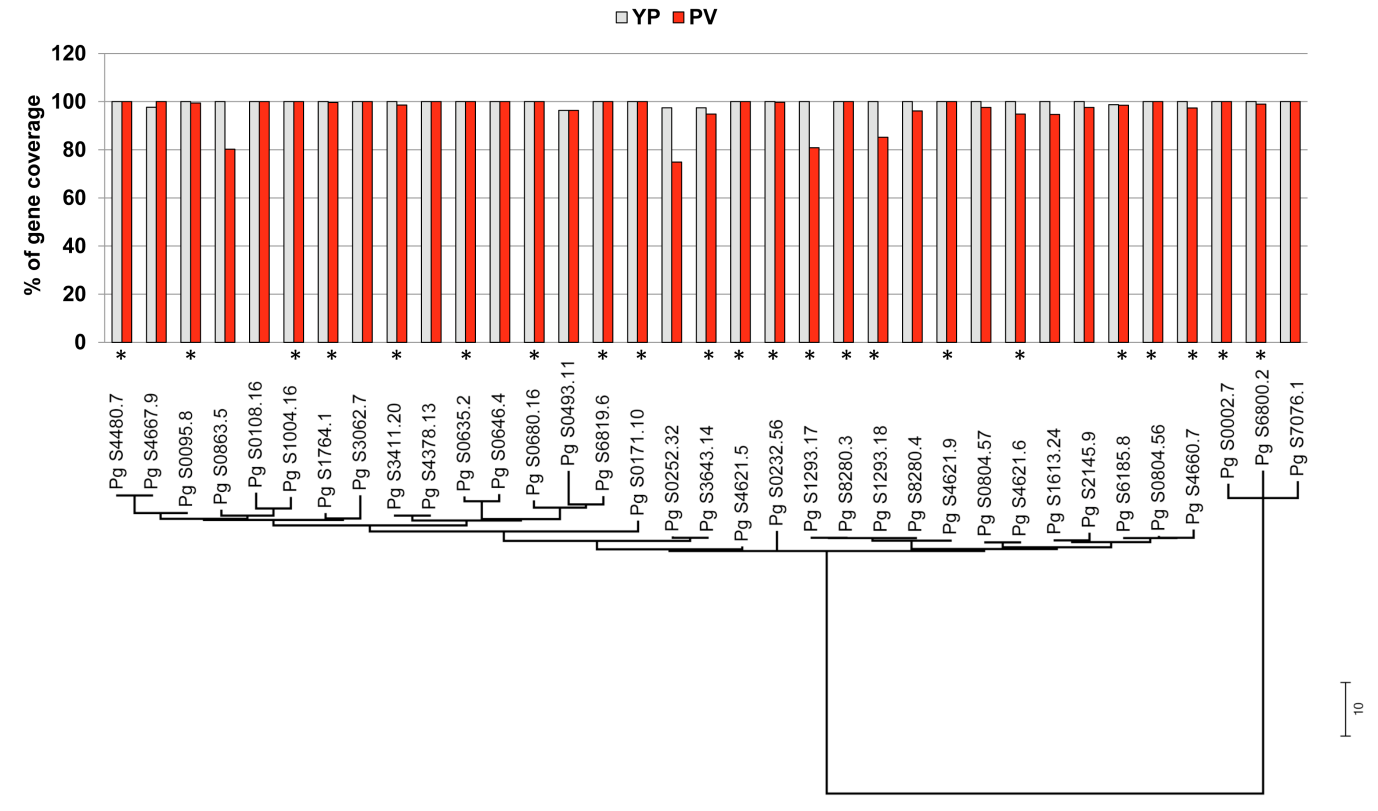


**Supplemental Figure 19. Classification and estimation of CAB orthologs gene copies.** The figures shows two layers of sub figures wherein the bottom figure represents the phylogenetic tree of CAB orthologs and the middle shows their corresponding coding (CDS) gene coverage using 10x WGS reads from *P. ginseng* cv. YuP (white bar) and diploid *Panax* species, *P. vietnamensis* (red bar)*.* In addition, orthologous CAB in *P. notoginseng* (PN) which is also a diploid was denoted as * under the bar graph

**Supplemental Figure 20. *P. ginseng* specific expansion of TF family genes.** Gene numbers of TF families identified in *P. ginseng* draft genome were compared with other plant species and amplification ratios of *P. ginseng* TF family genes were determined. The ratios were indicated by different colors for 3.0 or higher (yellow to red) and 2.0 or less (blue to green). TF families in red indicate that gene members in those families were expanded more than 3 times compared to other plant species. Dc, carrot; Euasterids, average gene number of 6 species belonging to Eusterids; Asterids, average gene number of 7 species belonging to Asterids; all, average gene number of 18 plant species. Refer to Supplementary note and **Supplementary Table 24** for detailed information. The highly expanded TF families were known to be involved in phyA-signaling (FAR1), GA response, cell differentiation, and dormancy induction (HRT), freezing tolerance (CSD), calcium signaling (CAMTA), ABA-signaling (TUB), abiotic stress response (HSF), auxin response (HB-PHD), basal transcription (BBR-BPC), and two-component system for various signaling (GARP-ARR-B).


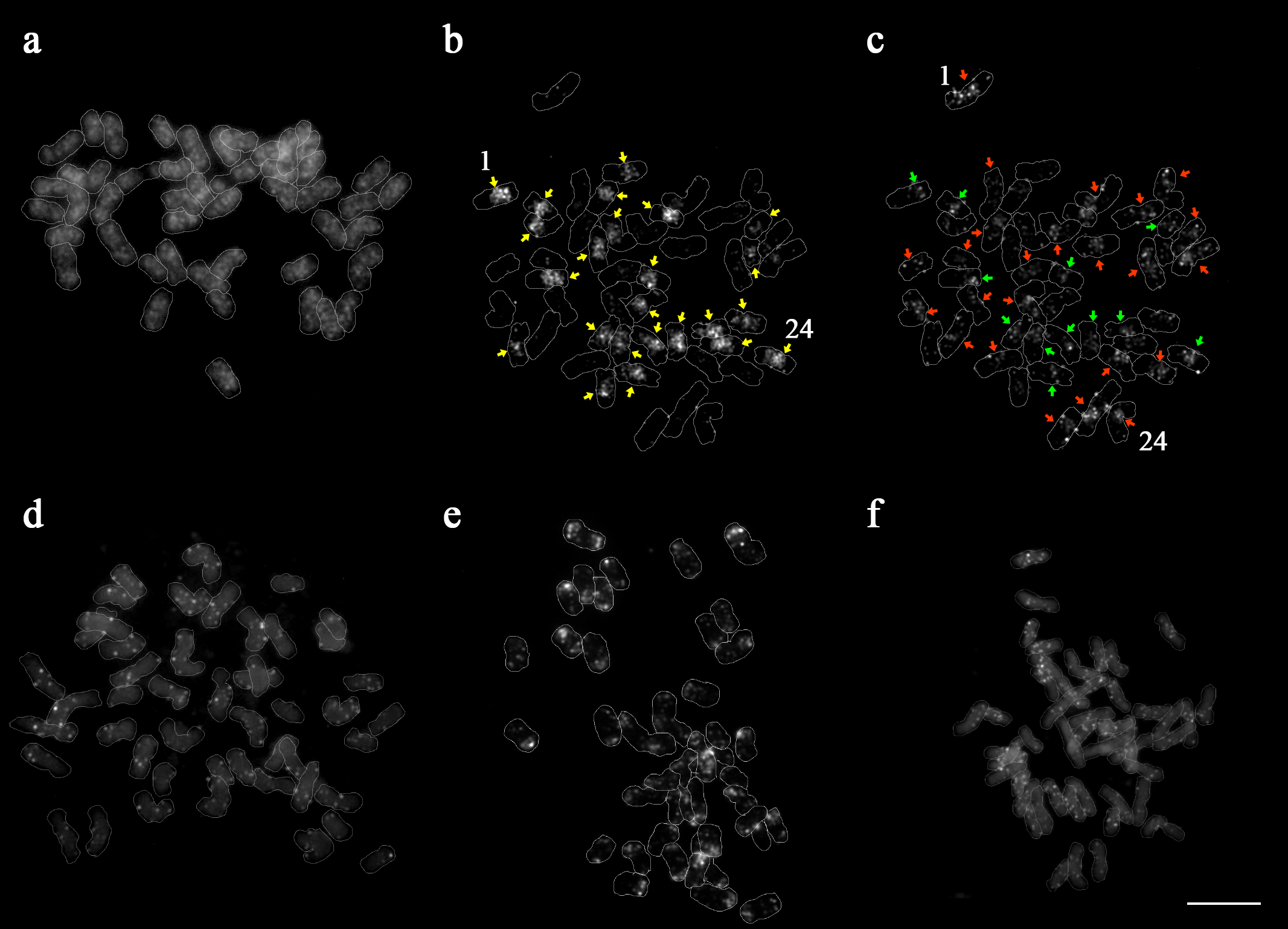


**Supplemental Figure 21. Chromosomal distribution of major *P. ginseng* REs in *P. ginseng* chromosomes.** Different repeat families hybridized to different chromosomal regions. **a**) *PgDel1* localized to all chromosomal regions, **b**) *PgDel2* shows strong signals in 24 of 48 chromosomes (yellow arrows indicate chromosome with intense *PgDel2* signals), **c**) *PgDel5* signals in *PgDel2* poor chromosomes (red and green arrows show *PgDel5* intense and *PgDel2-PgDel5* colocalization sites, respectively), **d**) *PgTork* in interstitial regions, **e**) *PgTat1* in subtelomeric regions, and **f**) *PgTat2* in pericentromeric regions. Bar = 10 µm.

**
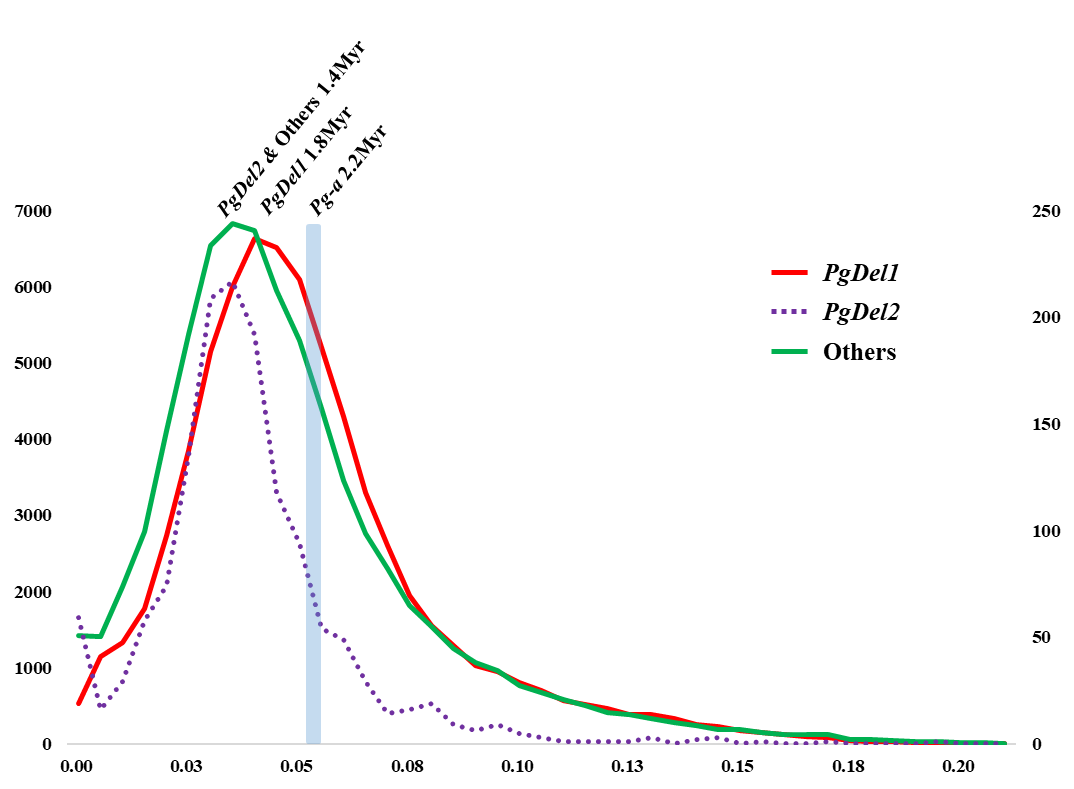
**

**Supplemental Figure 22.** **Estimation of LTR-RT insertion time in *P. ginseng***. The insertion time was calculated via comparison of LTR sequences of left and right LTRs for the relatively intact LTR-RTs. Comparison include 41,731 of *PgDel1* LTR-RTs, 5,090 of *PgDel2* LTR-RTs, and the other 67,301 LTR-RTs. Y-axis on the left indicate numbers of *PgDel1* and other LTR-RTs. Y-axis on the right indicate number of *PgDel2* LTR-RTs. A blurred blue box indicate time point of *Pg-α.*
